# From Current Therapeutics to Multitarget Ligands: A Review of Diabetes Pharmacological Treatments

**DOI:** 10.3390/pharmaceutics17091125

**Published:** 2025-08-28

**Authors:** Francesc Cabré, Josep J. Centelles, Marta Cascante

**Affiliations:** 1 Department of Biochemistry & Molecular Biomedicine, University of Barcelona, 08028 Barcelona, Spain; josepcentelles@ub.edu; 2 Medical Area, Menarini Group, 08918 Badalona, Spain; 3 Institute of Biomedicine of University of Barcelona (IBUB), University of Barcelona (UB), 08028 Barcelona, Spain; 4 CIBER of Hepatic and Digestive Diseases (CIBEREHD), Institute of Health Carlos III (ISCIII), 28029 Madrid, Spain

**Keywords:** diabetes, insulin resistance, review

## Abstract

Diabetes is a chronic and complex pathological syndrome that includes a series of disorders and imbalances, whose first characterization is hyperglycemia, although, as it is a multifactorial phenomenon, it requires risk reduction strategies beyond glycemic control. Continuous education and support for diabetes self-management are essential to prevent acute complications and reduce the risk of long-term complications. Therefore, the guidelines for the treatment of diabetes emphasize the importance of lifestyle changes, including a reduced-calorie diet and increased physical activity. However, for many people, these changes can be difficult to maintain in the long term and eventually they must resort to pharmacological treatment that in most cases requires the combined use of two or more antidiabetic drugs with different mechanisms of action. This review explores the different pharmacological agents, authorized and used therapeutically, for the control of diabetes, especially type 2 diabetes, and analyzes the development strategies of multi-target agents whose effects, through distinct mechanisms and by acting on more than one receptor, could represent a promising alternative in the treatment of a multifactorial disease such as diabetes. As regards therapeutic uses, from metformin to glucose transporter inhibitors (SGLT2i), the potential mechanisms of action, pharmacological and clinical effects, safety, and use in therapeutics are described, presenting, as far as reasonably possible, diverse comparisons between them. In conclusion, although metformin remains the first-line agent for the treatment of type 2 diabetes, the choice of a second-line agent depends on several factors, in particular the cardiovascular risk profile, weight, and renal function of the patient; moreover, the ideal pharmacological treatment, although expected and desired, has in fact not been achieved so far, and physicians must consider not only the glycemic efficacy of the agent but also all the other potential benefits, balanced by the possible adverse effects. Compounds modulating multiple signaling pathways are a promising approach to manage this multifactorial disorder, with the primary objective of maintaining the therapeutic efficacy observed in several clinical studies, alongside reducing adverse effects, the main reason for the discontinuation of developments, to levels that enable a favorable risk–benefit balance.

## 1. Diabetes and Insulin Resistance

### 1.1. A Multifactorial Scenario

Diabetes mellitus (DM) is a global epidemic that poses a significant threat and considerable risk to global health. According to estimates by the International Diabetes Federation (IDF), in 2024 [1], there were 588.7 million people from this pathology worldwide, and these values are expected to reach 852.5 million by 2050, DM generates a risk of early mortality, so the IDF estimated that over 3.4 million people aged 20–79 died as a result of DM and related complications in 2024. The number of children and adolescents (up to 19 years old) living with diabetes increases annually. Direct health expenditures due to diabetes surpassed one trillion USD and will continue to rise over the coming years. Type 2 DM (T2D) accounts for approximately 90% of all DM cases, and the global exponential growth of this epidemic is mainly attributed to sedentary lifestyles, obesity, population aging, urbanization, and economic development. Type 1 diabetes is the major type of diabetes in childhood; in 2024, over 1.81 million children and adolescents, younger than 20 years, had type 1 diabetes, but it can occur at any age, it cannot be prevented, and patients with type 1 diabetes require insulin to survive [1].

Diabetes is a chronic and complex pathological condition that includes a series of disorders, most of them characterized by hyperglycemia, but it requires continuous medical attention with multifactorial risk reduction strategies beyond glycemic control. Ongoing diabetes self-management education and support is critical to preventing acute complications and reducing the risk of long-term complications. Another cause for alarm is the consistently high percentage (45%) of people with undiagnosed diabetes, which is overwhelmingly type 2. This highlights the urgent need to improve the ability to diagnose people with diabetes, many of whom do not know they have diabetes, and provide appropriate and timely care to all people with diabetes as soon as possible [1,2,3].

The two main types of diabetes, type 1 (T1D) and type 2 diabetes (T2D), are classified on the bases of several parameters: age of onset, loss of β-cell function, insulin resistance, presence of autoantibodies associated with diabetes, and need for treatment with insulin [4]. Thus, T1D is caused by autoimmune destruction of β cells, which generally leads to absolute insulin deficiency, including latent autoimmune diabetes of adulthood, while T2D is caused by a progressive loss of adequate insulin secretion by β cells, often in the context of insulin resistance. There are other specific types of diabetes due to various causes, i.e., monogenic diabetes syndromes (neonatal diabetes and maturity-onset diabetes of the young), diseases of the exocrine pancreas (cystic fibrosis and pancreatitis), drug- or chemical-induced diabetes (corticosteroids, HIV treatment, post-organ transplant therapy), and gestational diabetes mellitus (diabetes diagnosed in the second or third trimester of pregnancy that was not clearly evident diabetes before pregnancy) (Table 1) [3,5].

Diabetes types 1 and 2 represent heterogeneous conditions characterized by substantial variability in clinical manifestation and disease trajectory. Accurate classification is essential for guiding therapeutic decisions; however, in certain cases, individuals may not be distinctly categorizable as either type 1 or type 2 at the time of diagnosis. The conventional view that type 2 diabetes is exclusive to adults and type 1 to children has become outdated, as both forms of the disease are now recognized across all age groups (Table 1) [2].

Another approach to classifying diabetes is based on the type of patients, and it consists of stratifying adult patients into distinct subgroups using a data-driven approach. This strategy leverages the most clinically relevant and readily accessible variables to identify subtypes that reflect the underlying pathophysiological mechanisms of the disease: GADA (glutamate decarboxylase antibodies), BMI (body mass index), HbA1c (glycosylated hemoglobin), age of onset of diabetes, homeostatic models to evaluate β-cell functionality (HOMA2-B), and resistance to insulin (HOMA2-IR) from fasting glucose and C-peptide. The selection of these variables is grounded in the understanding that diabetes arises when insulin secretion fails to meet physiological demands, often due to insulin resistance. This classification framework relies on indicators of both insulin secretion and insulin sensitivity, recognizing that hyperglycemia may result either from a primary deficiency in insulin production, as seen in type 1 diabetes (T1D), or from varying degrees of insulin resistance combined with inadequate compensatory insulin secretion, characteristic of type 2 diabetes (T2D). Five groups are considered that show different clinical characteristics and present a different progression of the pathology, as well as the derived complications: severe autoimmune diabetes (SAID), severe insulin-resistant diabetes (SIRD), severe insulin-deficient diabetes (SIDD), moderate obesity-related diabetes (MOD), and moderate age-related diabetes (MARD) [9].

The aim of this classification is to ensure that the resulting subtypes can improve the clinical approach, with a better prediction of the risk of complications and the response to therapy, as well as to facilitate greater power and specificity in scientific efforts by allowing studies in more homogeneous groups of patients.

Diabetes can be diagnosed based on plasma levels of glycosylated hemoglobin (A1C) or glucose. In the second case, plasma glucose values can be determined under fasting conditions (FPG); in an oral glucose tolerance test (OGTT) at 2 h (2-h PG) after ingesting 75 g of this monosaccharide; or by the random glucose value accompanied by classic hyperglycemic symptoms or hyperglycemic crises. Random is any time of the day without considering the time elapsed since the previous meal. In general, and summarizing it in a few lines, if possible, the diagnostic criteria for diabetes are FPG ≥ 7.0 mmol/L, or 2-h PG ≥ 11.1 mmol/L, or A1C ≥ 48 mmol/mol (65%), and in women, in nonpregnant individuals. Individuals with symptoms of hyperglycemia or hyperglycemic show a random plasma glucose ≥ 11.1 mmol/L [10].

### 1.2. Pathophysiology of Type 2 Diabetes Mellitus

The pathophysiology of type 2 diabetes mellitus (T2D) is collectively governed by several factors, with peripheral insulin resistance and pancreatic β cell dysfunction being the central aspects. Metabolic dysregulation contributing to hyperglycemia involves multiple mechanisms, including reduced insulin secretion, impaired glucose uptake, and increased hepatic glucose production. These alterations can lead to pathophysiological changes across various organs and systems. This metabolic imbalance is frequently associated with hypertension and dyslipidemia, characterized by elevated levels of low-density lipoprotein (LDL) and reduced levels of high-density lipoprotein (HDL) in serum, both of which significantly elevate cardiovascular risk (CV).

Metabolic dysregulation that contributes to hyperglycemia includes decreased insulin secretion, impaired glucose utilization, or increased glucose production and eventually causes pathophysiological changes in multiple organs and systems. It is often accompanied by hypertension and dyslipidemia: elevated serum concentrations of low-density lipoproteins (LDL) and low serum concentrations of high-density lipoproteins (HDL) that increase cardiovascular (CV) risk.

The exact molecular mechanism that leads to insulin resistance is not yet defined, so it is always concluded that the pathophysiology of diabetes responds to a highly complex and multifactorial system in which a multitude of processes, organs, and mediators are involved.

**Pancreas**. Insulin resistance in muscle and liver, as well as alteration of insulin secretion by β pancreatic cells, can be considered as initial premises that are the central defects of T2D. Two of the protagonists of this multifactorial system are located in the pancreas: insulin-producing β cells and α cells where the glucagon is synthesized. Glucagon is a very relevant mediator, a peptide catabolic hormone that promotes gluconeogenesis and glycogenolysis, i.e., it stimulates glucose production mainly in the liver. A second relevant peptide hormone is glucagon-like peptide-1 (GLP-1), an incretin hormone produced in the gut as a response to food and that increases insulin secretion. Resistance of β cells to GLP-1 contributes to progressive β-cell dysfunction, while increased glucagon levels and increased liver sensitivity to glucagon contribute to excessive glucose production by the liver [11,12,13,14].

**Skeletal muscle**. The action of insulin on muscle cells (myocytes) is a switch to promote the utilization and storage of glucose. Although the physiological outcomes (glucose uptake and glycogen synthesis) have been studied for a long time, their molecular basis is still unknown, with the involvement of a wide variety of protein mediators. In general, insulin, by activating its receptors (INSR) in myocytes, stimulates glucose uptake by vesicles containing the glucose transporter protein GLUT4 (GSV), which generates an increase in the intracellular production of glucose-6-phosphate that, together with coordinated dephosphorylation, allows the net synthesis of glycogen [15].

To define the scenario in a simple way, insulin resistance consists of two very well coupled mechanisms: the inability to interrupt glucose production and the lack of glucose uptake by peripheral tissues, mainly muscle. In an insulin-sensitive myocyte, activation of the INSR triggers the signaling cascade to increase glucose uptake and glycogen synthesis. Myocytes with insulin resistance are deficient in signal transmission, which reduces insulin’s ability to stimulate the GLUT4 transporter and glycogen synthesis. Skeletal muscle usually uses more than 80% of circulating glucose in the presence of insulin, in a normal situation, i.e., in healthy individuals, while in conditions of insulin resistance this effect is diminished [15].

**Liver**. The main direct actions of insulin on liver cells or hepatocytes, as in other types of cells, are carried out via activation of the INSR receptor. One of the most important actions is to stimulate glycogen synthesis and regulate gluconeogenesis by controlling hepatic glucose production. As mentioned above, the action of glucagon, a pancreatic hormone that stimulates the production of glucose, is especially relevant in the liver, where its effect is conveniently counteracted by insulin.

Insulin also has direct hepatocellular effects on lipid metabolism, the most prominent being the transcriptional upregulation of several genes involved in de novo lipogenesis (DNL), although it has also been described as causing an increase in the elimination of triglyceride-rich lipoproteins and a decrease in the export of very low-density lipoproteins (VLDL). The overall effect is to promote lipid storage in the hepatocyte and decrease the availability of fatty acids for oxidation by other tissues.

In the liver, insulin resistance, along with insulin deficiency, hyperglucagonemia, increased sensitivity to glucagon, and increased circulating levels of glucose precursors (fatty acids, lactate, glycerol, and amino acids), leads to increased gluconeogenesis, which is responsible for increased basal rate of glucose production and fasting hyperglycemia. Glucose production is a multifactorial process where glucagon plays a fundamental role. In fact, higher fasting glucagon concentrations correlate closely with increased fasting hepatic glucose production. A gradual increase in insulin resistance requires a markedly increased amount of insulin to overcome hyperglycemia, resulting in a complex interaction between insulin secretion and insulin resistance in the liver and skeletal muscle [14,15].

**Adipose tissue**. The cells of white adipose tissue, adipocytes, are extremely sensitive to the action of insulin, whose main physiological function in these cells is the suppression of lipolysis. Blocking lipolysis prevents the increase in plasma levels of free or non-esterified fatty acids (NEFAs). Downregulation of NEFAs is critical for maintaining normal glucose status (euglycemia). In adipocytes, insulin resistance and inflammation lead to synthesis and release of NEFA and pro-inflammatory cytokines, such as interleukin-6 (IL-6) or tumor necrosis factor (TNF). These mediators aggravate insulin resistance in the liver and muscle, contributing to β-cell dysfunction. Adipokines, cytokines secreted by adipose tissue, which sensitize insulin, such as adiponectin, on the other hand, counteract insulin resistance [14,15].

**Gastrointestinal system**. After eating, insulin secretion increases, and glucagon secretion is inhibited by the combined actions of hyperinsulinemia and hyperglycemia caused by incretin hormones. Approximately 60 to 70% of insulin secretion depends on the release of incretins, mainly glucagon-like peptide 1 (GLP-1) and glucose-dependent insulinotrophic polypeptide, also called gastric inhibitory polypeptide (GIP). GLP-1 is secreted by the L cells of the distal small intestine and GIP by the K cells of the more proximal small intestine [14,16]. These peptides have been shown to delay gastric emptying, stimulate insulin, and suppress glucagon secretion in a glucose-dependent manner.

In fact, the decrease in the “incretin effect” has been repeatedly demonstrated in patients with T2D [17,18]. In this sense, GLP-1 deficiency can be observed in individuals with glucose intolerance and progressively worsens with progression towards T2D. In addition to GLP-1 deficiency, there is resistance to the stimulatory effect of GLP-1 on insulin secretion. Unlike GLP-1, plasma GIP levels are elevated in T2D, but circulating plasma insulin levels are reduced, a consequence of β-cell resistance to the stimulatory effect of GIP on insulin secretion. Clearly, the intestine is an important endocrine organ and contributes to the pathogenesis of type 2 diabetes [14,16].

**Renal System**. In healthy adults, all filtered glucose is reabsorbed in the renal proximal tubule (~180 g/day). In the basolateral part, glucose leaves the cells following its concentration gradient mainly through the glucose transporter GLUT2 and re-enters the bloodstream. More than 90% of this reabsorption is a consequence of the action of the sodium-glucose cotransporter type 2 or sodium-glucose-linked transporter type 2 (SGLT2), which is expressed in the nearby renal proximal tubule, while the remaining filtered glucose is reabsorbed via the type 1 cotransporter (SGLT1), expressed in the descending part of the proximal tubule. Under normal physiological conditions, SGLT2 and SGLT1 are responsible for all renal glucose reabsorption. Maximum renal glucose transport is achieved when blood glucose levels exceed 11.1 mmol/L and excess glucose is eliminated in the urine. This renal safety valve can prevent extreme hyperglycemia [19].

In T2D, the maximal reabsorption capacity of the renal tubule is increased due to markedly elevated levels of SGLT2 mRNA and the protein itself in proximal renal tubule cells. Therefore, increased renal glucose reabsorption by the action of SGLT2 and increased threshold for urinary glucose elimination contribute to the maintenance of hyperglycemia. In other words, overactivation of an adaptive response of the kidney to conserve glucose appears to play an important role in the development of hyperglycemia in T2D [20,21].

**Central Nervous System**. The brain has an obligatory need for glucose and is responsible for 50% of glucose utilization under basal or fasting conditions. This glucose demand is met primarily by glucose production by the liver and, to a lesser extent, by renal reabsorption [16].

In fact, insulin secreted from the pancreas crosses the blood–brain barrier and binds to insulin receptors expressed throughout the central nervous system. Brain insulin signaling participates in a complex multiorgan process in the regulation of appetite, lipolysis, triglyceride secretion and absorption, thermogenesis, and hepatic glucose production. All of this ultimately protects the body from ectopic lipid deposition and hyperglycemia.

Overnutrition rapidly promotes insulin resistance in the brain, long before insulin signaling in peripheral organs is affected, suggesting that insulin resistance in the brain represents an early and important mechanism for dyslipidemia and hyperglycemia in the condition of hypernutrition.

Certain hypothalamic areas, those where appetite is regulated, show a decreased inhibitory response in individuals with T2D compared to normal glucose-tolerant subjects, although the plasma insulin response was observed to be markedly increased in obese subjects [22].

Finally, under stress conditions, the brain demands energy from the body through a mechanism known as “cerebral insulin suppression,” which enables it to meet its heightened energy requirements. In this context, the brain’s alternative strategies to safeguard its energy supply may contribute to the development of diseases such as obesity or type 2 diabetes mellitus. This theory originally named “selfish brain theory” [23,24] is based on the brain’s regulation of ATP levels, where ATP-sensitive calcium channels are thought to mediate the brain’s self-allocation of glucose. In other words, these compensatory mechanisms may lead to metabolic disorders like obesity or type 2 diabetes mellitus. Alternatively, in conditions of food abundance, an inefficient cerebral energy attraction could result in excessive accumulation, ultimately culminating in obesity and type 2 diabetes. Unlike traditional models, this theory alters the hierarchy of regulated parameters by prioritizing the brain’s energy demands. Consequently, adipose tissue size or muscle mass becomes a secondary regulatory target. According to this framework, obesity and type 2 diabetes mellitus are conceptualized as brain-related disorders involving defects in neuroendocrine function [23,24]. In support of this hypothesis, a model on centralized anaerobic–aerobic energy balance compensation providing a theoretical explanation has been recently reported [25].

**Figure 1:**
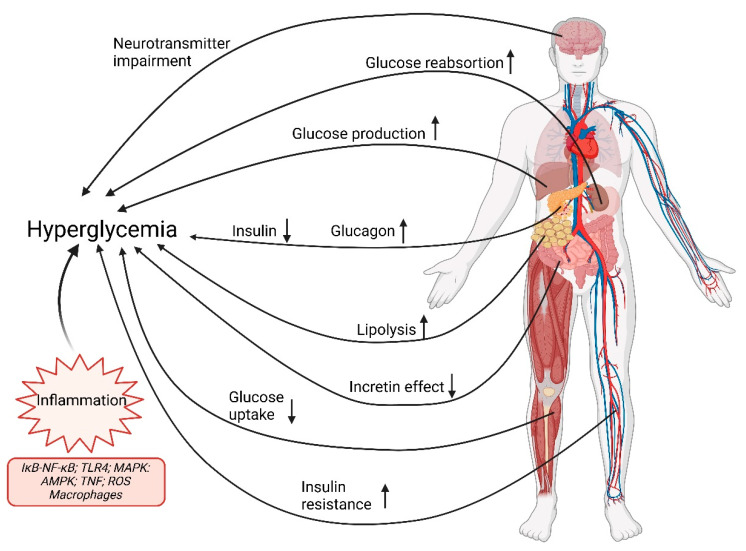
Key organs and systems of hyperglycemia in type 2 diabetes (Created in BioRender. Cascante, M (2025), https://BioRender.com/v70l924 (accessed on 27 July 2025)). Key aspects in type 2 diabetes mellitus: **insulin resistance** in **muscles** and **liver** and **deficient insulin secretion** by pancreatic β cells. Mechanisms involved: progressive **failure of β-cell** function due to resistance to glucagon-like peptide 1 (GLP1); **excessive production of glucose** by the liver caused by **increased levels of glucagon** and greater hepatic sensitivity to glucagon; **insulin resistance in adipocytes accelerates lipolysis** and increases levels of plasma free fatty acids (FFAs), which aggravate insulin resistance in muscles and liver and contribute to β cell failure; **increased renal glucose** reabsorption by sodium/glucose cotransporter 2 (SGLT2) maintains hyperglycemia; **resistance to the appetite-suppressing effects** of insulin, leptin, GLP1, amylin, and peptide YY, as well as low levels of brain dopamine and elevated levels of brain serotonin contribute to weight gain, exacerbating the underlying resistance; **vascular insulin resistance**; **proinflammatory** cytokines induce insulin resistance.

**Summary**. It is complex to summarize the broad scenario that governs the pathophysiology of type 2 diabetes mellitus (T2D), or the multiorgan functional alterations that insulin resistance entails (Figure 1). These pathologies are associated with multifunctional alterations: insulin secretion is altered; fasting plasma glucagon levels increase and are not normally suppressed after a meal; basal hepatic glucose production increases and fails to be suppressed normally after a meal; muscle glucose uptake is impaired; fasting plasma free fatty acid levels increase and are not normally suppressed after a meal; and the increase in GLP-1 and GIP after meals is normal or slightly decreased [14].

Insulin resistance in muscle and liver and impaired insulin secretion by pancreatic β cells are the core of T2D defects. β-cell resistance to glucagon-like peptide 1 (GLP-1) contributes to their progressive dysfunction, while increased glucagon levels and increased liver sensitivity to this hormone contributes to excessive glucose production by the liver.

In adipose tissue, insulin resistance leads to enhanced lipolysis and elevated plasma free fatty acid (FFA), which further impair insulin sensitivity in muscle and liver and contribute to hepatic dysfunction of β cells.

Moreover, increased renal glucose reabsorption facilitated by sodium-glucose cotransporter 2 (SGLT2) and a higher renal threshold for glucose excretion contribute to the maintenance of hyperglycemia.

A set of resistances, such as to the appetite-suppressing effects exerted by insulin; to adipokines, such as leptin which sensitize insulin; to the peptide hormone GLP-1 that increases insulin secretion; and to a set of regulators, as well as low levels of brain dopamine and increased levels of brain serotonin, contribute to weight gain. Everything together, in turn, exacerbates the underlying insulin resistance. To all this must be added inflammation and vascular resistance to insulin.

As a relevant aspect, it should be noted that this pathology is strongly associated with both micro- and macrovascular complications. Therefore, cardiovascular (CV) diseases, especially heart failure (HF), have the greatest impact on mortality in the diabetic population. The close association between T2D and heart failure is a result of the detrimental effect of the key pathogenic factors: chronic glucotoxicity and lipotoxicity, as well as impaired insulin signaling.

## 2. Treatment of Type 2 Diabetes Mellitus

The scenario described above with multifactorial pathophysiological alterations shows the complexity of the management of T2D. It is a very important medical problem due to its increasing global prevalence and because chronic hyperglycemic states are closely related to obesity, liver disease, and several cardiovascular diseases. As a first consideration, the essential concepts in diabetes management can be mentioned: age, body weight, complications, duration, education, cost, and etiology—concepts that aim to guide doctors in the use of therapeutic agents in a more effective, efficient and safe way [26]. Prevention of microvascular complications focuses on glycemic control, while prevention of macrovascular complications requires correction of the classic cardiovascular risk factors that make up the insulin resistance syndrome.

The first step in defining or characterizing T2D is typically based on one or more of the following diagnostic criteria: impaired fasting glucose (IFG), impaired glucose tolerance (IGT), or elevated glycated hemoglobin A1c (HbA1c) levels. Individuals with IFG are obviously characterized by elevated fasting plasma glucose levels. IGT is characterized by insulin resistance in the muscle and impaired late (second phase) insulin secretion after a meal. For their part, individuals with altered plasma glucose levels, i.e., IFG, exhibit hepatic insulin resistance and impaired early insulin secretion (first phase) (Table 2).

Although, strictly speaking, there is currently no cure for T2D, a major lifestyle change minimizes risk factors; glycemic control is mandatory, and the need for antidiabetic drugs, to reverse the pathophysiological effects, is constant. Just to mention one example, the US Food and Drug Administration (FDA) has approved more than 60 antihyperglycemic drugs since the approval of human insulin in 1982, more than half of them are new chemical entities (NCEs) administered as monotherapy, with the rest being unique combinations of two or more antihyperglycemic agents [27]. These drugs are grouped into different classes: insulins, biguanides (metformin), sulfonylureas, meglitinides (glinides), α-glucosidase inhibitors, thiazolidinediones (TZDs), incretin-dependent therapies, and sodium/glucose cotransporter type 2 inhibitors (SGLT2i). Newer drugs, which target established molecular pathways, such as glucagon-like peptide-1 (GLP-1) receptor agonists, dipeptidyl peptidase 4 (DPP-4) inhibitors, and SGLT2i, have gained popularity in the pharmaceutical market, while less expensive over-the-counter alternatives are increasing in developing economies. Furthermore, since no single medication reverses multiple abnormalities, combination therapy has gained wide acceptance and will continue to grow. New combinations containing, for example, metformin, SGLT2i, and DPP-4 inhibitors are seeing considerable use in the last decade, and approvals of combination regimens targeting multiple pathways for T2D control have been increasing. Finally, the great heterogeneity of T2D is also creating the need to promote more personalized and accessible treatments.

## 3. Metformin

Although the biguanide-type compound metformin was first introduced in 1959, its widespread clinical adoption occurred two decades later in the United States. It was approved by the US Food and Drug Administration (FDA) on 29 December 1994 and, based on promising trial data, it became the first-line therapy for the management of T2D in 2005 [28]. In contrast, other biguanides such as phenformin and buformin were withdrawn from clinical use due to their association with a high incidence of lactic acidosis [29,30].

Metformin is currently the most widely prescribed antidiabetic agent globally. Its primary mechanism of action involves the suppression of hepatic glucose production, resulting in reduced fasting plasma glucose and HbA1c levels. However, it does not directly affect pancreatic β-cell function and, in the absence of weight loss, does not significantly enhance insulin sensitivity in skeletal muscle. Consequently, after an initial improvement, HbA1c levels tend to rise progressively over time. Although the precise mechanisms by which metformin inhibits hepatic glucose output remain incompletely understood, proposed pathways include the inhibition of key glycolytic and gluconeogenic enzymes, as well as mitochondrial glycerophosphate dehydrogenase. These effects are thought to initiate a cascade of biochemical events, beginning with the activation of AMP-activated protein kinase (AMPK) [31]. This cascade ultimately leads to reduced plasma glucose and lactate concentrations and diminished hepatic gluconeogenesis [27,32,33,34]. Recent hypotheses suggest that metformin may also exert its effects through the stimulation of cyclic phosphatidylinositol phosphate (PIP) synthesis—a natural antagonist of cyclic AMP—which may trigger additional insulin-regulatory pathways [35].

The most common adverse effects of metformin are gastrointestinal: nausea, indigestion, abdominal cramps or bloating, diarrhea, or some combination of these, most of which decrease over time with continued use of the drug and can be minimized by starting with low doses and increasing gradually up to a target dose. Metformin is associated with a 20–30% reduction in blood levels of vitamin B12, probably due to poor absorption. Although the precise biological mechanism remains unclear, several studies and meta-analysis reported correlation between prolonged metformin use and reduced serum levels of vitamin B12, indicating that individuals undergoing metformin therapy tend to exhibit lower concentrations of this vitamin. Given these findings, routine monitoring of vitamin B12 levels has to be implemented in patients receiving long-term metformin treatment, particularly those with over five years of exposure or presenting with peripheral neuropathy [36]. Furthermore, metformin has also been associated with low-incidence lactic acidosis (3–6 per 100,000 patient-years) [37]. In a relatively old clinical study conducted in the UK and in the USA, cardiovascular events were significantly reduced in a small group of 344 obese patients with diabetes who were treated with metformin [38]. There is no consensus on renal contraindications, but metformin should be discontinued preventively in situations where renal function may decline.

## 4. Action of Insulin Secretion

### 4.1. Sulfonylureas

Sulfonylureas (Table 3) were considered as the second class of oral hypoglycemic agents. In fact, they constituted the second line to use if metformin monotherapy did not work adequately. However, current consensus and guidelines place sulfonylureas below GLP-1-RA or SGLT2i in most scenarios because sulfonylureas lack cardio-renal benefit and carry a higher hypoglycaemia risk [6]. The most used oral hypoglycemic drug was glipizide, followed by glimepiride and then sitagliptin [39].

The history of sulfonylureas began with the hypoglycemic activity of synthetic sulfur compounds (p-amino-sulfonamide-isopropylthiodiazoles), used as antibiotics in patients with typhoid fever who experienced a hypoglycemic effect after treatment [41].

Sulfonylureas reduce plasma glucose levels by increasing insulin secretion from the pancreas. They bind to a transmembrane receptor (sulfonylurea receptor) that modulates ATP-sensitive K^+^ channels (KATP) in the cell membrane of pancreatic β cells, causing depolarization and opening of Ca^2+^ channels. This effect results in hyperinsulinemia that suppresses insulin resistance, decreases fasting plasma glucose and HbA1c levels. However, after the initial decrease, HbA1c progressively increases because sulfonylureas do not have a long-term protective effect on β-cell function and could accelerate the failure of β-cell function [40].

Sulfonylureas commonly cause hypoglycemia and are associated with weight gain, and some retrospective studies suggest that they may increase cardiovascular events, including being associated with increased cardiovascular mortality. The different types of sulfonylureas (Table 3) differ in their selectivity for β cells and their risk of cardiovascular effects. Compared to glibenclamide (glyburide), the most favorable profiles have been observed with gliclazide followed by glimepiride and glipizide. Gliclazide is apparently safer, it has been associated with a reduced risk of cardiovascular mortality, and it is less likely to cause weight gain and hypoglycemia [5,40,42].

Less common side effects of sulfonylureas include nausea and vomiting, cholestatic jaundice, agranulocytosis, aplastic and hemolytic anemias, generalized hypersensitivity reactions, and dermatological reactions [5].

The gradual addition of sulfonylurea to metformin, or vice versa, is associated with progressive failure of β-cell function and an increase in HbA1c. Newer hypoglycemic agents, with less risk of hypoglycemia or with proven cardiovascular benefits, prioritize sulfonylureas. However, because the low treatment cost, metformin and sulfonylureas remain the most prescribed oral antidiabetic agents worldwide [5].

### 4.2. Glinides or Meglitinides

Meglitinides, also known as glinides (repaglinide, nateglinide, and mitiglinide), are short-acting insulin secretion stimulators that help the pancreas to produce insulin by closing the same K^+^ channels as sulfonylureas, but at a different binding site. By closing the K^+^ channels of pancreatic β cells, they open the Ca^2+^ channels, thus increasing insulin secretion [43,44,45]. Unlike sulfonylureas, meglitinides, particularly nateglinide, exhibit a glucose-sensitive action, so their potency increases at high glucose concentrations [43,46]. Meglitinides are short-acting; are associated with lower risks of hypoglycemia, weight gain, and chronic hyperinsulinemia than sulfonylureas; and require administration before each meal. However, they do not prevent the progressive decline in β-cell function and increase in HbA1c that is associated with T2D. Additional studies have shown that meglitinides may be associated with an increased risk of hypoglycemia in diabetic patients with advanced chronic kidney disease [47].

The FDA authorized two meglitinides: nateglinide in 2009 and repaglinide in 2013. In January 2001, the EMA authorized Prandin^®^ (repaglinide) of Novo Nordisk A/S. There are currently several generics of repaglinide in Europe. On 29 April 2022, the European Commission withdrew the marketing authorization for Starlix^®^ (nateglinide), at the request of the authorization holder, Novartis Europharm Limited, which notified its decision to permanently discontinue marketing the product for commercial reasons. Currently, there are no meglitinides in clinical trials [27].

## 5. Insulin Sensitizers

### Thiazolidinediones (Glytazones)

Thiazolidinediones (TZDs), strictly speaking, can be considered the only insulin-sensitizing agents. They enhance or improve the action of insulin in various tissues and organs: skeletal muscle, cardiac muscle, liver, and adipocytes, and they exert a powerful effect on pancreatic β cells to increase and preserve insulin secretion. It is suggested that TZDs may have a similar but longer-lasting effect than metformin, which is explained by their insulin-sensitizing action together with the stimulation of β cells [48].

Multiple mechanisms mediate the insulin-sensitizing effects of TZDs, with particularly relevant stimulation of peroxisome-proliferator-activated receptor γ (PPARγ), increased insulin signaling, and stimulation of various stages of glucose metabolism. In addition, participation has also been demonstrated in the increase in lipid oxidation and redistribution, proliferation of subcutaneous adipocytes, activation of genes involved in lipogenesis, reduction of plasma levels of free fatty acids (FFAs), and reduction of circulating inflammatory cytokines, etc. [49,50,51].

Troglitazone was the first TZD authorized by the FDA in 1997, but it was discontinued in 1999 due to severe hepatotoxicity [52]. The next two TZDs were rosiglitazone and pioglitazone, both approved by the FDA in 1999, and by the EMA in 2000. Rosiglitazone has been associated with an increased risk of myocardial infarction and was withdrawn in some countries. In 2010, the FDA published a safety alert about cardiovascular risks, although it did not recommend its withdrawal and it is still available in the USA. In Europe, in September 2010, the EMA recommended suspension of rosiglitazone because the benefits no longer outweighed the risks [53,54]. Pioglitazone favorably affects many components of the insulin resistance syndrome, and a reduction in myocardial infarction, stroke, and cardiovascular death was observed [55].

Adverse events of TZDs, such as increased fat mass and trauma-related fractures in postmenopausal women, are dose-dependent, and doses above 30 mg/day should be avoided. Weight gain is common with TDZ and correlates with decreased HbA1c, improvements in insulin sensitivity, and β-cell function. TZDs cause or exacerbate congestive heart failure in some patients. After initiation, and after dose increases, patients should be carefully observed for signs and symptoms of heart failure (including excessive and rapid weight gain, dyspnea, and/or edema). If signs and symptoms develop, discontinuation or dose reduction should be considered. Concerns about bladder cancer were allayed in a review of approximately one million individuals from six populations, where no increase in bladder cancer was observed with pioglitazone or rosiglitazone [50,56].

Overall, TZD prescription rates are experiencing a gradual decline, likely due to reports of safety concerns. However, each territory handles the situation differently due to income levels, degrees of drug development, and physiological differences in the population [27]. Pioglitazone is, as mentioned above, the most used TZD, and in several territories the only one, occupying its place in all available diabetes management guidelines as the first or second option. A combination regimen consisting of pioglitazone and metformin is also currently marketed.

## 6. Incretin-Dependent Therapies

Incretins are a family of peptide hormones that are produced in the intestine in response to food ingestion. They are responsible for 70% of total insulin secretion after oral administration of glucose and, as mentioned above, the main ones are glucagon-like peptide 1 (GLP-1) and the glucose-dependent insulinotrophic polypeptide (GIP). These hormones are secreted by the L cells of the distal small intestine (GLP-1) and by the K cells of the more proximal small intestine (GIP) [14,16].

GLP-1, the most potent of the incretins, stimulates insulin release from pancreatic β cells in a glucose-dependent manner, facilitating reuptake and metabolism of glucose in muscle and lipolysis in fatty tissue. On the other hand, it reduces pancreatic production of glucagon, leading to a decrease in hepatic glucose production. In addition, it delays gastric emptying, causing a decrease in postprandial hyperglycemia; decreases appetite; and increases the feeling of satiety, which leads to a reduction in body weight and reduces plasma glucagon (Table 4) [57,58]. GLP-1 release is accentuated by oral glucose administration, and T2D patients exhibit altered GLP-1 secretion postprandially, which may explain the inadequate insulin response [57,58,59,60].

Native circulating GLP-1 acts on the pancreas in 2–4 min as it has a short plasma half-life due to its rapid degradation by the enzyme dipeptidyl peptidase 4 (DPP-4). Generally, this cleavage has no adverse effects on glucose levels; on the contrary, this inactivation is necessary. However, in cases of T2D, the production of incretins is reduced and their effects are weakened, and moreover, their rapid inactivation does not allow these hormones to perform their physiological functions, leading to hyperglycemia in patients with T2D [17,61].

The development of potential antidiabetic drugs with the aim of enhancing or maintaining the physiological effect of incretins has been approached from two different pharmacological strategies: agents that mimic the action of incretins for a long time by activating their receptors, GLP-1 receptor agonists, and those that prevent the destruction of incretins by blocking the action of the enzyme that cleaves them (DPP-4) with DPP-4 inhibitors.

### 6.1. GLP-1 Receptor Agonists (GLP-1 RA)

The starting point in the design of pharmacological agents that act as GLP-1 modulators was the natural peptide exendin 4. This peptide, originally isolated from the saliva of the lizard Heloderma suspectum, although it shares only 53% structural homology with native GLP-1, activates the GLP-1 receptor with the same affinity as the natural ligand, but is resistant to degradation by DPP-4. Therefore, the goal is synthetic peptides with a strategic sequence to avoid degradation by DPP-4 but maintaining sufficient homology to activate the GLP-1 receptor with a potency equivalent to GLP-1 [62,63].

Synthetic GLP-1 peptide analogues with agonistic affinity for the GLP-1 receptor, such as albiglutide, dulaglutide, exenatide, liraglutide, lixisenatide, and semaglutide, are administered subcutaneously, and an oral formulation of semaglutide is also available; all of them are resistant to degradation by DPP-4 (Table 5). Based on a considerable number of clinical studies and results observed in routine clinical practice over 10 years, the efficacy and safety of GLP-1 RAs are well established. Data obtained with a wide variety of GLP-1RAs suggest that the results observed in clinical practice, including in the primary care setting, are consistent with those observed in clinical trial programs. There is also some evidence that the effectiveness of GLP-1 RA is maintained through long-term use in terms of glycemic control and body weight [64].

GLP-1 RAs cause a pharmacological increase in plasma GLP-1 levels, markedly increase insulin secretion, and inhibit glucagon secretion. Increased plasma insulin and decreased glucagon effectively suppress hepatic glucose production and cause a long-lasting reduction in HbA1c [65]. GLP-1 agonists are probably among the most effective classes of medications to reduce glucose [5,66].

**Table 5:** Overview of key features of some GLP-1 RA authorized by the EMA and/or FDA for the treatment of T2D [66].

Drug Route	Dosage Regime		Pharmaceutical Form
*Short-Acting*			
Exenatide SC	2/day Initial 5 µg After 1 month ↑ 10 µg based on clinical response	60 min before the two main meals. Interval ≥6 h	Pre-filled pen (glass) (10 and 5 μg)
Lixisenatide ^†^ SC	1/day Initial 10 µg 14 days Day 15 ↑ 20 µg	1 h before the 1st meal of the day	Cartridge in pre-filled pen (20 and 10 μg)
*Long-Acting*			
Dulaglutide SC	1/week Monotherapy 0.75 mg Add-on therapy 1.5 mg ↑ 1.5 mg if glycemic control is not adequate. Max 4.5 mg/wk	Anytime	Pre-filled pen (0.75, 1.5, 3, and 4.5 mg)
Exenatide XR SC	1/week 2 mg	Anytime	Powder in vial + solvent in pre-filled syringe (2 mg) Cartrige in pre-filled pen (2 mg)
Liraglutide ^‡^ SC	1/day 0.6 mg week 1 Day 8 ↑ 1.2 mg If after 1 week, inadequate glycemic control ↑1.8 mg	Anytime	Pre-filled pen (6 mg/mL, doses of 0.6, 1.2, or 1.8 mg administered)
Semaglutide SC	1/ week 0.25 mg 4 weeks week 5 ↑ 0.5 mg If no adequate glycemic control: 4 weeks later ↑ 1 mg; and 4 weeks later ↑ 2 mg	Anytime	Pre-filled pen (0.25, 0.5, 1, and 2 mg)
Semaglutide oral	1/day 3 mg 30 days Day 31 ↑ 7 mg If no adequate glycemic control: 30 days later ↑ 14 mg	30 min before the 1st meal, drink, or other medication of the day	Oral tablets (3, 7, and 14 mg)

Short-acting: Drug concentrations decline to low levels within a few hours after administration. Long-acting: Drug concentrations are maintained at effective concentrations throughout the dosing period. ^†^ Also available as a fixed-dose combination with insulin glargine 100 U; ^‡^ also available as a fixed-dose combination with insulin degludec 100 U. SC: Subcutaneous; wk: week, XR: Extended Release. The upward arrow [↑] indicates that, after the aforementioned time of treatment with the initial dose, it should be increased to the indicated dose.

Beyond glycemic control, GLP-1 RA promotes weight loss, which improves insulin sensitivity, delays gastric emptying, corrects endothelial dysfunction, reduces blood pressure, improves plasma lipid profile, and reduces C-reactive protein levels. Combination therapy with a GLP-1 RA plus basal insulin has been reported to be highly effective in reducing HbA1c and preventing weight gain associated with insulin therapy, without increasing the risk of hypoglycemia [67,68]. In addition, these drugs have shown beneficial effects concerning cardiovascular disease (CVD) prevention in T2DM in recent clinical trials; thus, in the 2023 European Society of Cardiology (ESC) guidelines for the management of CVDs in patients with diabetes, GLP-1 RA are indicated as the first-choice treatment for T2DM patients at a high/very high CV risk or those with proven atherosclerotic CVD [69].

As regards the effects on body weight, GLP-1 RA is currently the gold standard in the treatment of overweight patients with T2DM. However, data generated in the SELECT trial, a large scale multicenter clinical study to evaluate whether treatment with semaglutide reduces the risk of major cardiovascular events among patients with overweight or obesity who did not have diabetes, suggests that treating overweight or obese people with semaglutide would be another element in the prevention of secondary atherosclerotic cardiovascular disease, along with other standard evidence-based practices, such as pharmacological treatment of hypertension, diabetes, and dyslipidemia [70,71]. Semaglutide and liraglutide have therapeutic indications as a complement to a low-calorie diet and increased physical activity for weight control, including weight loss and maintenance, in adults who are obese or overweight, and with at least one comorbidity related to weight i.e., dysglycemia (prediabetes or T2D), hypertension, dyslipidemia, obstructive sleep apnea, or cardiovascular disease, and in 12 years or older adolescents with obesity or a weight greater than 60 kg, as a complement to a low-calorie diet and greater physical activity for weight control [72,73,74].

Clinical trials have shown that GLP-1 RA does not increase the risk of major adverse cardiovascular effects (MACE) (Table 6). Additionally, treatments with once-weekly subcutaneous dulaglutide, liraglutide, and semaglutide demonstrated significant reductions in the incidence of these effects compared with placebo. Therefore, dulaglutide, liraglutide, and semaglutide are indicated to reduce cardiovascular risk in patients with T2D and cardiovascular disease (CVD) or multiple cardiovascular risk factors (dulaglutide only) [5,66,75]. In addition, in clinical studies evaluating cardiovascular outcome, where renal outcome is also evaluated as a secondary endpoint, GLP-1 RAs have a beneficial effect on renal outcome compared to placebo [76,77].

The most common adverse events of GLP-1 RA are gastrointestinal, mainly nausea, vomiting, and diarrhea, and can sometimes be dose-limiting. Nausea and vomiting are quite common but are usually mild and disappear in 4 to 8 weeks [5].

The potential association between GLP-1RAs and tumor development remains a subject of ongoing debate. While some clinical trials have reported a higher incidence of malignancies in patients treated with semaglutide compared to control groups, real-world data on the oncologic safety of GLP-1RAs are still scarce. A pharmacovigilance study investigated this issue by analyzing reports from the FDA Adverse Event Reporting System (FAERS), identifying 8718 tumor-related cases potentially linked to GLP-1RA use [78]. After excluding pre-existing malignancies, concomitant use of other glucose-lowering agents, or unrelated adverse events, the analysis focused on cases where GLP-1RAs were the primary suspected drug. The findings did not indicate a disproportionate increase in overall cancer risk. However, notable safety signals were observed for specific tumor types, including thyroid carcinoma, particularly papillary subtype, malignant pancreatic neoplasms, and islet cell tumors. Interestingly, the co-administration of GLP-1RAs with DPP-4is may have contributed to the elevated reporting rates for certain neoplasms. This study offers new real-world insights into the oncologic safety of GLP-1RAs and underscores the importance of clinician awareness regarding potential tumor-related adverse effects, especially when these agents are used in combination with DPP-4is [78].

Postmarketing reports suggested an association between GLP-1 RA and the risk of pancreatitis, although there is currently insufficient data to establish whether this relationship is causal or not. In any case, GLP-1 RA should be avoided in patients with a history of pancreatitis. Cases of acute alterations in the gallbladder, such as cholelithiasis and cholecystitis, have been described with GLP-1 RA. If such events are suspected, discontinuing treatment should be considered. Finally, GLP-1 RAs have been associated with serious hypersensitivity reactions, including angioedema and anaphylactic reactions, but these events are rare [3,5,66].

Finally, a clinical trial of semaglutide reported higher rates of complications of diabetic retinopathy (vitreal hemorrhage, blindness, need for treatment with an intravitreal agent or photocoagulation) compared to placebo. However, it is important to note that 84% and 83% of these events occurred in patients with diabetic retinopathy at baseline, in the semaglutide and placebo groups, respectively. There were no unexpected safety signals for diabetic retinopathy in the other trials, and further analysis indicated that there was no increased risk among those patients without diabetic retinopathy at baseline [79,80].

### 6.2. Dipeptidyl Peptidase-4 Inhibitors (DPP-4i)

The second pharmacological strategy that allows prolonging the action of incretin hormones is DDP-4 inhibitors that prevent the rapid enzymatic degradation of GLP 1. These DPP-4 inhibitors (alogliptin, linagliptin, saxagliptin, sitagliptin, vildagliptin) prolong the half-life of endogenously secreted GLP-1 by blocking the enzyme responsible for the degradation of GLP-1 (DPP-4). Therefore, by preventing the degradation of GLP-1, DPP-4 inhibitors maintain adequate circulating levels of GLP-1, and the main effect of these agents in improving glycemic control is mediated by inhibition of glucagon secretion and the reduction of basal hepatic glucose production. It is a heterogeneous class of unrelated compounds, with different pharmacokinetic profiles (Table 7).

The potential benefits and risks of DPP-4i can be categorized into class effects, which occur as a consequence of the inhibition of DPP-4 activity, and compound-specific effects, related to the individual chemical entities.

The ability of DPP-4 inhibitors to increase insulin secretion and reduce HbA1c is modest (approximately 0.50% to 0.70%). An advantage of this class of drugs is that they do not cause hypoglycemia unless they are used in combination with other agents (sulfonylureas, insulin, etc.). Unlike GLP-1 agonists and SGLT2 inhibitors, DPP-4 inhibitors do not have a weight-reducing effect and are considered weight-neutral [3,5,58].

DPP-4 inhibitors have a largely neutral effect on cardiovascular outcomes. However, in clinical trials with saxagliptin and alogliptin, a certain risk of hospitalization for heart failure was observed in the group treated with the drug compared to the placebo group, and although the mechanism of heart failure with these drugs is unclear, the FDA suggests avoiding medications containing saxagliptin or alogliptin in patients with heart failure (Table 8) [58,81,82].

Certain pieces of evidence of the renoprotective effect with the use of DPP-4 inhibitors are under discussion: reduction of albuminuria in T2D patients with pre-existing albuminuria and potential reduction of the decrease in estimated glomerular filtration rate. Most DPP-4 inhibitors are eliminated renally (Table 7) and therefore require dose adjustment in CKD (chronic kidney disease), except linagliptin, which is mainly eliminated via the enterohepatic route, and can be used in patients with CKD without dose adjustment [5,83].

DPP-4 inhibitors have an excellent safety profile. They may cause some gastrointestinal side effects, although not to the extent that GLP 1 agonists do. Concerns about pancreatitis have not been substantiated in prospective trials, as the causal relationship has not been definitively established [84]. In postmarketing reports, DPP-4 inhibitors have also been associated with hypersensitivity reactions, including anaphylaxis and angioedema, and are therefore contraindicated in patients with a history of hypersensitivity reactions.

## 7. Action on Renal and Intestinal Glucose Absorption

### 7.1. α-Glucosidase Inhibitors (AGIs)

Before discovering and clarifying the endocrine pathophysiological role of the gastrointestinal system and the effect of incretins on the development of T2D, a class of drugs targeting the small intestinal α-glucosidase enzyme was introduced into clinical practice and, therefore, to decreased glucose absorption. The first drug in this category of α-glucosidase inhibitors (AGI) was acarbose, authorized by the FDA in 1995. Subsequently, in 1996, mitiglitol was authorized. An additional AGI, voglibose, was authorized by the Japan Pharmaceuticals and Medical Devices Agency in 1999 [27].

AGIs (acarbose, voglibose, miglitol) decrease the rate of carbohydrate absorption in the intestine and increase meal-stimulated GLP-1 secretion. These agents exert competitive and reversible inhibition of pancreatic α-amylase and/or membrane-bound intestinal α-glucosidase-type enzymes. Pancreatic α-amylase hydrolyzes complex starches to oligosaccharides in the lumen of the small intestine, while intestinal α-glucosidases hydrolyze oligosaccharides, trisaccharides, and disaccharides to glucose and other monosaccharides in the small intestine. In diabetic patients, these enzyme inhibitions result in a delay in glucose absorption and a decrease in postprandial hyperglycemia [85,86].

AGIs have been shown to have similar efficacy than metformin. For that reason, they are often prescribed as a first-line treatment or are combined with other antidiabetics, due to the modest impact on HbA1c (0.7–0.8%). In fact, their effect in lowering HbA1c is comparable to the effect of DPP-4 inhibitors [87,88,89]. The typical adverse effects of AGIs are gastrointestinal: flatulence, bloating and abdominal discomfort, diarrhea, nausea, vomiting, etc. It has been described in a meta-analysis that the use of AGIs leads to an increase in hepatic transaminases, indicative of hepatotoxicity [90].

### 7.2. Sodium-Glucose-Linked Transporter Type 2 Inhibitors (SGLT2i)

The renal proximal tubules are essential in the filtration and reabsorption of glucose and are responsible for the absence of glucose in the urine. Under normoglycemic conditions, in healthy subjects, the proximal tubules of the kidneys reabsorb all the filtered glucose (about 180 g/day), and when plasma glucose concentration exceeds 10 to 11.1 mmol/L, glycosuria is detected.

The proximal tubules are composed of three segments: S1, S2 and S3, which contain transport proteins essential to facilitate the transport of glucose to the renal tubule. Sodium-glucose-linked transporter (SGLT) proteins facilitate glucose reabsorption in the proximal renal tubule. In the basolateral membrane, the sodium-potassium transporter adenosine triphosphatase (Na^+^/K^+^ ATPase) actively drives Na^+^ from the tubular cells into the circulation; this situation generates a Na^+^ gradient, which allows SGLTs to passively transport glucose from the lumen to the cells following this Na^+^ gradient. There are two types of SGLT: type 2 (SGLT2), which is located in segment S1, and type 1 (SGLT1), which is found in segments S2 and S3 [19,91,92].

In conditions of normoglycemia and with the transporters not altered, the type 2 transporter (SGLT2) is responsible for practically 97% of fractional glucose reabsorption in the entire kidney. In comparison, the type 1 transporter (SGLT1) contributes to reabsorbing only the remaining 3% of filtered glucose. Glucose transporters GLUT-1 and GLUT-2 located on the basolateral membrane transport glucose from tubular cells into the circulation [19,91].

In patients with T2D, the glycemic deprivation causes the kidneys to retain more glucose. As a result, the glucose reabsorption capacity and the glycosuria threshold increase. Furthermore, lack of glucose supply to cells leads to upregulation of SGLT2 for increased retention [92]. The relevance of SGLT2 in glycemic regulation has been demonstrated in non-clinical studies, such as obese Zucker rats, one of the most classic animal models in the experimental study of diabetes. These rats show an increase in the expression of the messenger RNA (mRNA) of SGLT2, probably to manage the increase in filtered glucose load in diabetes. Furthermore, induction of diabetes with streptozotocin in SGLT2 knockout mice resulted in a smaller increase in blood glucose level compared to wild-type mice [93].

Inhibitors of sodium-glucose-linked transporter (SGLT2i) are a new class of antidiabetic drugs that mediate epithelial glucose transport in the renal proximal tubules, inhibiting glucose absorption, resulting in glycosuria, and therefore, they improve glycemic control [91].

The capacity of the SGLT1 transporter to reabsorb glucose is manifested when the SGLT2 transporter is blocked or during hyperglycemia, a capacity that in normoglycemic conditions could reach up to 40–50%. As a consequence, diabetes-induced hyperglycemia or SGLT2 inhibition would increase induced glucose transport facilitated by the SGLT1 transporter, which is the rational basis for dual inhibition of SGLT1/2 (see below) [19].

SGLT2 inhibitors (canagliflozin, dapagliflozin, empagliflozin, ertugliflozin, ipragliflozin, luseogliflozin, tofogloflozin, etc.) block glucose absorption in the proximal renal tubule. They decrease the renal glucose reabsorption capacity and reduce the blood glucose threshold at which glucose is eliminated in the urine (glycosuria). That is, the kidney can release more glucose. Increased removal of glucose from the body through glycosuria leads to a reduction in plasma glucose, resulting in decreased glucotoxicity, with improved β-cell function and increased insulin sensitivity.

FDA and EMA have approved four oral SGLT2i agents for the treatment of T2D: canagliflozin, dapagliflozin, ertugliflozin, and empagliflozin (Table 9). Ipragliflozin, luseogliflozin, and tofogliflozin have been approved in Japan. Remogliflozin was first commercially launched in India. Development of sergliflozin was discontinued after phase II trials [92].

SGLT2 inhibitors modestly improve hemoglobin A1c (HbA1c) in patients with T2D but have additional benefits for weight loss and blood pressure reduction, due to calorie loss in the urine. Additionally, SGLT2 inhibitors resulted in a lower risk of hypoglycemia compared to agents such as sulfonylureas or insulin [5,95]. SGLT2i has also demonstrated cardiovascular benefits, especially in the treatment of heart failure in both diabetic and non-diabetic populations, which has made them the preferred hypoglycemic agents to treat T2D patients at high risk of cardiovascular events [96,97,98]. Dapagliflozin and empagliflozin already have the therapeutic indication, in addition to T2D, for heart failure and chronic kidney disease.

The cardiovascular endpoint in clinical trials demonstrated cardiovascular safety but did not demonstrate superiority in cardiovascular outcomes. However, in trials involving high-risk patients with established cardiovascular disease at baseline, empagliflozin and canagliflozin showed a reduction in the primary composite outcome of cardiovascular death, nonfatal myocardial infarction, and nonfatal stroke. Although some clinical trials were not specifically designed to evaluate renal outcome, when evaluated as a secondary outcome, the possible benefit of empagliflozin and canagliflozin was suggested [99,100,101].

In cardiovascular or trials, SGLT2 inhibitors are associated with a 30–35% lower risk of heart failure hospitalization [100,101,102,103]. Other hypoglycemic agents appear to be more potent than SGLT2 inhibitors but fail to reduce cardiovascular risk, particularly with respect to heart failure outcomes. In addition, although the hypoglycemic efficacy of SGLT2 inhibitors decreases with low estimated glomerular filtration rates (eGFR), the cardiovascular benefits are remarkably preserved, even in patients with renal failure (Table 10). This implies different mechanisms of action in controlling glycemia and reducing cardiovascular risk as well as cardioprotective mechanisms that are not completely understood. Some authors suggest that a combination of systemic and direct effects of SGLT2 inhibition on the myocardium ultimately leads to cardiovascular benefits [96].

Adverse effects include genital fungal infections in female patients, balanitis in uncircumcised male patients, urinary tract infections, and side effects in older patients and people taking diuretics. Postmarketing data of acute kidney injury, probably secondary to intravascular volume depletion, has been reported. On the bases of data currently available, the FDA recommends evaluation and monitoring of kidney function in patients with risk factors for kidney injury, such as those with preexisting chronic kidney disease, congestive heart failure, or taking medications such as diuretics or antihypertensives [5].

One safety concern of SGLT2 inhibitors is related to the risk increase in euglycemic diabetic ketoacidosis (DKA). The incidence rate of DKA was approximately 0.5 per 1000 patient years in people taking SGLT2 inhibitors compared with 0.2 per 1000 patient years in people taking other agents. For this reason, off-label use of SGLT2 inhibitors is discouraged in people with T1D. However, the evidence in T2D is controversial; for example, in a meta-analysis of 10 randomized clinical trials involving 13,134 patients and 14 DKA events, overall event rates were 0.1% in the group of patients treated with SGLT2 inhibitors versus 0.06% in the control groups [104]. Thus, SGLT2 inhibitors were not associated with a significantly increased risk among T2D patients. The authors are cautious, however, and state that they cannot rule out the possibilities of a modest effect on DKA by SGLT2 inhibitors, an effect on a specific clinical phenotype of DKA (e.g., euglycemic DKA), or an unclassified effect. Therefore, they justify greater monitoring to resolve the uncertainty about this specific safety problem of this type of drug. However, a recent meta-analysis provides reassuring data on the safety of SGLT2i (dapagliflozin and empagliflozin) use, particularly in children and young people with T2D and T1D. Although it is possible that the increased urinary glucose excretion caused by SGLT2i may cause urinary tract infections, the incidence was similar compared to placebo, and although β-hydroxybutyrate levels were significantly higher in the SGLTi group compared to placebo on T1D, the elevation was mild and not severe enough to cause DKA [105].

In a clinical trial of canagliflozin, excess risk of amputation was observed, predominantly at the toe or metatarsal level, compared with placebo. The underlying pathophysiology is not well understood. However, data observed in clinical practice suggests that the risk of amputation is probably not a class effect because empagliflozin and dapagliflozin were not associated with an excess rate of amputation [106].

## 8. Multitarget Ligand Strategies

Despite the wide range of available antidiabetic medications, monotherapy with individual drugs often fails to adequately control blood glucose levels and address associated comorbidities. As a result, treatments involve combination therapies that utilize drugs with different mechanism of action. However, this multidrug approach can lead to challenges such as increased risk of side effects, toxicity, and undesirable drug–drug interactions. Moreover, patient adherence to these complex regimens tends to be low, although it could potentially be improved by co-formulating multiple active ingredients into a single tablet. An emerging alternative is the development of single compounds capable of simultaneously modulating multiple therapeutic targets, offering a more balanced profile of efficacy and safety compared to conventional single-target treatment.

### 8.1. Dual Glucose-Dependent Insulinotropic Polypeptide (GIP) and GLP-1 Receptor Agonist

In May and September 2022, the FDA and EMA, respectively, approved tirzepatide [107,108,109], a dual GIP and GLP-1 receptor agonist. This drug was given once a week by subcutaneous injection to manage blood glucose levels in adults with type 2 diabetes (T2D), complementing a balanced diet and regular physical activity. Phase III clinical trials showed that tirzepatide achieved superior glycemic control compared to placebo [110,111], weekly subcutaneous semaglutide at 1.0 mg [85], insulin degludec [112], insulin glargine [113], and insulin lispro [114]. Beyond glycemic benefits, tirzepatide also improved liver fat levels and reduced both visceral and subcutaneous abdominal fat volumes [115]. A comprehensive meta-analysis found that tirzepatide outperformed other long-acting GLP-1 receptor agonists and other comparators in lowering blood glucose and body weight. However, its use was linked to a higher frequency of gastrointestinal side effects, particularly nausea [116]. The Summary of Product Characteristics (SmPC) of tirzepatide includes similar warnings and precautions as those listed for other GLP-1 receptor agonists. Moreover, clinical trial data indicate that tirzepatide does not elevate the risk of major cardiovascular events [117]. Recent reviews exploring its cardioprotective potential suggest that tirzepatide can help lower cardiovascular risk factors in people with T2D by reducing blood pressure, HbA1c, and body weight. It has also shown positive effects on cardiometabolic risk markers in individuals with obesity who do not have diabetes [109,118,119]. Two large cardiovascular outcome trials are ongoing in patients with T2DM (SURPASS-CVOT) and in patients with obesity (SURMOUNT-MMO) [120].

The safety profile of tirzepatide closely resembles that of GLP-1RAs, with gastrointestinal adverse events being the most commonly reported. While clinical trials and observational studies involving GLP-1RAs have raised concerns regarding potential risks such as pancreato-biliary complications, diabetic retinopathy, and medullary thyroid carcinoma, current evidence regarding these outcomes in tirzepatide-treated patients remains limited and inconclusive. A systematic review and meta-analysis encompassing nine randomized clinical trials demonstrated that tirzepatide does not confer an elevated risk of pancreatitis compared to basal insulin, placebo, or selected GLP-1 RAs [121]. However, an increased incidence of composite gallbladder and biliary tract disorders was observed when compared to basal insulin or placebo. Notably, within the SURPASS clinical program, which included 9687 participants, only a small number of pancreato-biliary adverse events were reported [120].

Postmarketing surveillance data from the FDA Adverse Event Reporting System (FAERS) have identified signals related to gastrointestinal and pancreato-biliary disorders, diabetic retinopathy, and medullary thyroid cancer. In this analysis, tirzepatide exhibited a comparable risk of gastrointestinal adverse events and medullary thyroid carcinoma to GLP-1RAs, while showing a reduced risk for most pancreato-biliary complications and diabetic retinopathy. Overall, the safety profile of tirzepatide appears consistent with that of GLP-1RAs, without a discernible increase in the aforementioned risks [122]. Ongoing large-scale trials, including SURPASS-CVOT and SURMOUNT-MMO, are expected to provide further clarity and validation of these findings

### 8.2. GLP-1 and Glucagon Dual Agonists

The peptide glucagon is potentially involved in appetite suppression and in energy expenditure increase facilitating weight reduction. On the basis of that, the combination of glucagon and incretin hormones could produce enhanced, synergistic benefits in the treatment of diabetes and other metabolic disorders [123,124]. In fact, some studies demonstrated that the simultaneous infusion of GLP-1 at low doses and glucagon can suppress appetite and boost energy expenditure, as was observed when each peptide was administered individually [125].

Molecules that can simultaneously target both the GLP-1 receptor (GLP-1R) and the glucagon receptor are being explored, and first examples of peptides functioning as dual agonists were reported. In animal models of diet-induced obesity (DIO), treatment with these co-agonists resulted in more significant reductions in body weight and lipid levels compared to single-agonist therapies, all without inducing hyperglycemia or other adverse effects [126,127], and peptides with balanced activation on both receptors delivered the most favorable outcomes in terms of weight loss and blood glucose regulation [128]. However, due to the peptide condition one essential point to take into account to improve their therapeutic potential is evaluate the pharmacokinetic characteristics, and it remains crucial to thoroughly assess the safety profile of these compounds, particularly concerning long-term administration and potential cardiovascular side effects.

As an example, it is interesting to mention the development of analogues of oxyntomodulin (OXM), a gut hormone of 37 amino acids released after food intake, and like GLP-1, it exerts its effects through simultaneous activation of both the GLP-1 and glucagon receptors. However, one of the main limitations of OXM is its short plasma half-life—approximately 12 min—due to rapid degradation by the enzyme DPP-4, which cleaves the peptide’s first two N-terminal amino acids. To counteract this instability and maintain therapeutic efficacy, modified versions of OXM have been explored, particularly with alterations at the N-terminus to resist DPP-4-mediated breakdown. In preclinical experiments, these engineered compounds demonstrated glycemic control similar to traditional GLP-1R agonists. Importantly, they also promoted weight loss and reduced food intake, offering a potential promising advantage for obesity and diabetes treatment [129,130].

### 8.3. GIP, GLP-1, and Glucagon Multiple-Agonists

Some peptides have been designed to exhibit effects not only equivalent to incretin hormones but also including those of glucagon, since levels of incretins and glucagon rise significantly after bariatric surgery, and this physiological response associated with marked metabolic improvements is not totally achieved with current treatments [131,132].

Given that GLP-1, GIP, and glucagon share similar N-terminal sequences, this structural overlap facilitates the creation of peptides able to activate multiple receptors. A crucial aspect in developing these multi-receptor agonists is calibrating their relative activity at each receptor.

For instance, YAG-glucagon, a glucagon-based triagonist, was shown to significantly improve blood glucose levels in mice fed a high-fat diet but had no impact on body weight, probably due to different activity profiles in the receptors when using concentrations below the maximum [133]. To address these limitations, analogues are being developed. One of them, NN1706 (MAR423 or RO6883746), is a fatty-acylated tri-agonist that activates GLP-1R and glucose-dependent insulinotropic polypeptide receptor (GIPR) in a balanced way, while showing comparatively reduced activity at the glucagon receptor. In preclinical models—including obese mice, rats, and non-human primates—NN1706 administration led to notable reductions in body weight and enhanced glycemic control. In clinical trials involving overweight or obese individuals, once-daily subcutaneous dose induced significant, dose-related weight loss without affecting glycemic regulation. Nevertheless, an increase in heart rate was consistently observed in the NN1706-treated group, raising concerns that may limit its future clinical development. [134]

### 8.4. SGLT-1/SGLT-2 Inhibitors

As previously discussed, the glucose-lowering effect of SGLT2 inhibitors is partially offset by increased glucose reabsorption via the SGLT1 transporter, which can reclaim approximately 30–40% of filtered glucose once SGLT2 is inhibited. This has led to growing interest in dual inhibition of both SGLT2 and SGLT1 as a potential therapeutic strategy. When SGLT2 is inhibited, even partial (around 30%) inhibition of SGLT1 has been shown to boost urinary glucose excretion by up to 80%. Additionally, inhibiting intestinal SGLT1 by 30% produces an effect similar to that of acarbose, resulting in further reductions in HbA1c levels by approximately 0.5–0.6% [19,92,135]. Sotagliflozin, the most advanced dual SGLT1/SGLT2 inhibitor developed to date, encountered setbacks during phase III trials in patients with type 2 diabetes (T2D) and heart failure, largely due to financial challenges and disruptions caused by the COVID-19 pandemic [136]. Initially, the FDA declined approval for its use in combination with insulin for type 1 diabetes (T1D) [92]. However, development was continued, and in May 2023, the FDA granted approval for sotagliflozin to reduce the risk of cardiovascular death and hospitalization for heart failure in adults with heart failure, T2D, chronic kidney disease, and other cardiovascular risk factors [136,137]. In Europe, the EMA granted marketing authorization for sotagliflozin in April 2019 for use as an adjunct to insulin therapy to improve glycemic control in adults with type 1 diabetes mellitus; but on 22 March 2022, the European Commission withdrew the marketing authorization in the European Union. The withdrawal was at the request of the marketing authorization holder, Guidehouse Germany GmbH, which notified the European Commission of its decision not to market the product in the EU for commercial reasons [138].

### 8.5. Dual Inhibitors of Aldose Reductase (AR) and Protein Tyrosine Phosphatase 1B (PTP1B)

Emerging therapeutic strategies are also exploring novel molecular targets such as aldose reductase (AR) and protein tyrosine phosphatase 1B (PTP1B), two enzymes that play pivotal roles in the pathogenesis and progression of T2DM and its associated complications. AR, a key enzyme in the polyol pathway, contributes to the overproduction of intracellular reactive oxygen species (ROS) in various tissues (including the heart, vasculature, kidneys, and eyes), potentially driving the development of diabetes-related complications. On the other hand, PTP1B is known to negatively regulate insulin and leptin signaling and has been implicated in the development of insulin resistance and obesity [139]. 4-Thiazolidinone derivatives have been synthesized aimed at inhibiting both AR and PTP1B [140]. Although these compounds currently demonstrate significantly greater potency against human AR, ongoing efforts are focused on optimizing their chemical structures to achieve a more balanced dual-inhibition profile.

### 8.6. PPAR-α/γ Dual Agonists

Given that T2DM often coexists with cardiovascular risk factors such as hypertension, dyslipidemia (including elevated triglycerides and cholesterol), and hyperuricemia, there is increasing interest in comprehensive treatment approaches. As a result, current research is also targeting broader metabolic dysfunctions and their regulatory mechanisms. Within this framework, the design and development of dual agonists for peroxisome proliferator-activated receptors (PPARs) has emerged as a particularly promising avenue [141].

Peroxisome-proliferator-activated receptors (PPARs) are a family of nuclear receptor proteins encoded by distinct genes, consisting of three main subtypes: PPAR-α, PPAR-δ (also referred to as PPAR-β), and PPAR-γ. Each subtype exhibits a unique tissue distribution. PPAR-α, in particular, is predominantly found in tissues characterized by high rates of fatty acid oxidation, such as the liver, kidneys, cardiac muscle, and vascular endothelial cells. Activation of PPAR-α enhances lipid metabolism, which in turn leads to increased levels of high-density lipoprotein cholesterol (HDL-C) in the plasma.

In addition to its metabolic effects, PPAR-α was evidenced to exert certain anti-inflammatory and anti-atherogenic actions, primarily through the downregulation of inflammatory mediators and adhesion molecules [142,143]. Long-term administration of PPAR-α agonists was associated with reduced lipotoxicity and inflammation, resulting in improved cardiac function in diabetic individuals and a notable decrease in cardiovascular risk factors commonly associated with diabetes [144].

The development strategy of dual PPAR agonists was designed to achieve a combined and synergistic effect as antidiabetic and cardioprotective agents, given their potential efficacy in glycemic control through activation of one receptor, and lipid regulation through activation of the other. This approach represented a step beyond that of selective PPAR agonists. Among the various approaches explored, dual PPAR-α/γ agonists yielded the most promising results, demonstrating therapeutic potential in the treatment of diabetes, in cardiovascular protection, and in the management of dyslipidemias. Clinical studies with these dual agents showed significant reductions in triglyceride levels along with increased HDL cholesterol concentrations, which exert cardioprotective effects. These metabolic improvements are accompanied by enhanced insulin sensitivity, supporting their potential for the simultaneous treatment of glycemic and lipid abnormalities in patients with type 2 diabetes mellitus (T2DM) [145,146,147,148].

However, despite the relatively long list of dual agonists at different stages of development (aleglitazar, chiglitazar, imiglitazar, leglitazar, lobeglitazone, muraglitazar, naveglitazar, netoglitazone, saroglitazar, ragaglitazar, tesaglitazar, etc.), their safety has remained a subject of continuous debate for nearly two decades, and the clinical development of many of them has been discontinued due to the emergence of significant adverse effects. Only chiglitazar in China (October 2021) lobeglitazone in the Republic of Korea (July 2013), and saroglitazar in India (June 2013) have been approved for T2DM treatment. As an example, saroglitazar, the dual PPARα/γ agonist approved in India for the treatment of diabetic dyslipidemia, in clinical studies appeared to be well tolerated with a favorable safety profile [147,148].

Despite the adverse effects observed with PPAR dual agonists, particularly with certain PPARα/γ, which led to the discontinuation of their development, research on these dual-acting compounds remains ongoing. The primary objective is to retain their therapeutic potential in the treatment of T2DM and dyslipidemia while minimizing adverse effects to achieve a reasonably acceptable benefit–risk balance. Several compounds are currently in the early stages of development; however, further studies are clearly needed to accurately assess both their therapeutic efficacy and safety profiles.

## 9. Insulin

As described and referred above, in physiological situations, glucose is normally controlled by the coordinated secretion of two hormones, insulin and glucagon, which exert opposite functions to maintain glucose within the appropriate physiological levels required by the organism. Insulin is a two-chain polypeptide hormone, produced and released by pancreatic β cells and used as an essential treatment in T1D and T2D.

The secretion of endogenous insulin, under basal conditions (fasting and sleeping), is continuous to maintain sufficient glucose levels. In general, secretion is lowest in the middle of the night and highest in early morning; in fasted situations, it suppresses glycogenolysis and stimulates gluconeogenesis and lipogenesis. On the other hand, insulin secretion is also modulated by nutrient intake, which responds in a biphasic secretion manner: a rapid first phase followed by a slower and more sustained second phase. As already mentioned, the incretin hormone system contributes significantly to insulin secretion via GLP-1 and GIP, which stimulate insulin and suppress glucagon release from the pancreas, providing an increase in insulin secretion. Endogenous insulin is secreted into the portal venous system and then the liver eliminates at least 50% of the released insulin. The remaining hormone moves into the systemic circulation where it acts on its receptors, giving rise to a sequence of biochemical processes that trigger cellular responses such as glucose uptake, glycogen synthesis, and lipogenesis [15,149].

Exogenous insulin does not follow the same route as endogenous insulin, since, administered subcutaneously, it is distributed throughout the organism without first-pass hepatic uptake. Thus, with exogenous insulin the proportion of peripheral versus hepatic is much higher than with endogenous insulin. This altered relationship is the basis for the pharmacokinetics and additional side effects of exogenous insulin, in other words, the therapeutic use of either human insulin or analogues [15,149].

### 9.1. Insulin and Antidiabetic Therapy

For T1D, insulin is the primary treatment and is also indicated for patients with T2D diabetes who experience severe hyperglycemia (HbA1c > 10%), including a fasting glucose level greater than 250 mg/dL, which indicates glucose toxicity, in surgery to regulate glucose or during a hospitalization when oral medications are recommended to be discontinued and insulin becomes the preferred treatment. However, since insulin resistance is one of the key pathophysiological mechanisms of T2D, insulin treatment can still contribute to this resistance, which is why it should be used at key moments in diabetes, titrating in a timely manner and addressing each situation specifically.

Trials of combining insulin with other drugs to control T2D (GLP-1 RA, DDP-4i, and SGLT2i) suggest that such treatment could lead to the use of reduced doses of insulin, less weight gain, and fewer episodes of hypoglycemia compared with the insulin monotherapy. In fact, the combination of GLP-1 RA with basal insulin therapy causes a strong reduction in HbA1c and insulin dose reduction weight loss, and the combination of SGLT2i with insulin also effectively reduces HbA1c, insulin dose, and hypoglycemia and promotes weight loss [150].

Although it is well known that insulin is the main stay of T1D treatment, it may be difficult for some patients to achieve target HbA1c levels with insulin monotherapy. In some cases, hypoglycemia, excessive glucose fluctuations, and weight gain may also occur with intensive insulin therapy. Phase II–IV clinical trials are investigating the use of GLP-1 RA, DPP-4i, SGLT2i, selective SGLT1 inhibitors, and dual SGLT1/SGLT2 inhibitors as adjuvant insulin therapy for adult patients with T1D. Its place in therapy in the management of T1D remains to be defined [151].

Insulins used therapeutically can be classified according to their origin (human or modified analogues), which in all cases are biotechnological produced using recombinant RNA technology; depending on its pharmacokinetics, that is, onset of action, time when the maximum effect is reached and duration of action (ultra-rapid, rapid, intermediate, short-acting, prolonged, biphasic); or according to the mode of action and objective (basal, prandial, premixed, or mixtures).

### 9.2. Basal Insulins

Basal insulins simulate the insulin that is continuously secreted under physiological conditions to maintain glucose homeostasis in the fasting state (Table 11). They inhibit hepatic glucose production to keep the patient close to fasting normoglycemia. Basal insulin secretion accounts for approximately 50% of total 24 h insulin production. They are those that cover the insulin requirements between meals.

Basal insulin alone is the most convenient initial insulin regimen and can be added to metformin and other oral agents. Control of fasting glucose can be achieved with human NPH insulin or with the use of a long-acting insulin analog. Long-acting basal insulins (glargine U-100 or detemir) reduce the risk of symptomatic and nocturnal hypoglycemia compared with NPH insulin. Longer-acting insulins (glargine U-300 or degludec) may carry a lower risk of hypoglycemia compared to glargine U-100 when used in combination with oral agents

NPH (neutral protamine Hagedorn) [158] insulin or isophane human insulin is an intermediate-acting insulin, a modification of regular or human insulin that is obtained by adding protamine, in order to delay the onset of action and prolong the duration of the effect. It is obtained by mixing regular insulin and protamine in exact proportions with zinc and phenol, so that a neutral pH is maintained. Versions based on human and porcine insulin are available.

Human insulin is produced in Escherichia coli strains by recombinant DNA technology. The mixture of regular insulin (30%) and NPH insulin (70%) allows for a rapid onset of action and at the same time a prolonged duration of the effect, which can reach 24 h. It can be administered, mainly at night, in one or two doses, in combination with oral drugs. Although it does not adequately replicate the basal physiological pattern, due to its lower cost, it constitutes a good cost-effective option.

Detemir [159,160,161,162,163] is a recombinant analogue of insulin obtained from Saccharomyces cerevisiae. Less variable and with a more predictable profile than NPH insulin, this insulin analog has a 14C fatty acid, myristic acid, attached to Lys B29. The fatty acid side chain increases self-association and binding to albumin. This fact, together with slow systemic absorption from the injection site, prolongs tissue distribution, resulting in a long-lasting action. The duration of action depends on the dose: 12 h (0.2 IU/kg) and 20 h (0.4 IU/kg). In approximately one-third of patients, two doses must be administered to adequately cover 24 h. It usually requires higher doses (20–30% more) than glargine and NPH.

Glargine [60,63,64,65] is obtained by recombinant DNA technology in Escherichia coli or Pichia pastoris. It differs from endogenous human insulin by the replacement of Asn A21 by Gly and the addition of two Arg at the C terminus (positions B31 and 32). The resulting protein is soluble at pH 4 and forms microprecipitates at physiological pH (7.4), which allows the slow release of small amounts of insulin glargine, providing the drug with a long-lasting action and a non-pronounced maximum concentration. The onset of action is slower than that of human NPH insulin, and its action profile is prolonged, lasting up to 18–24 h. It has less variability in its absorption than NPH, so it better reproduces physiological basal insulin secretion. It should be administered once a day at any time, but every day at the same time, although it is preferable in the morning when nocturnal hypoglycemia appears. Once-daily injection of insulin glargine reaches steady-state levels 2–4 days after the first dose. The 300 IU/mL formulation represents a reduction in injection volume and a more sustained release with a flatter and more prolonged pharmacodynamic and pharmacokinetic profile.

Degludec [164,165] is an insulin obtained by recombinant DNA technology in Saccharomyces cerevisiae. Compared to endogenous insulin, it has an added hexadecanedioic acid at Lys B29 that facilitates the formation of multihexamers, resulting in a subcutaneous soluble depot, allowing stable secretion and a half-life greater than 24 h. The duration of action can reach 42 h with a variability four times less than that of glargine 100 IU, with the same effectiveness and lower rates of nocturnal hypoglycemia. It is the longest acting basal insulin. Daily administration and at the same day time are recommended, but its characteristics allow a high flexibility in the administration, with periods between two doses ranging from a minimum of 8 h to a maximum of 42 h.

To improve treatment adherence, different basal insulin formulations are being developed, aimed to have a long half-life and a prolonged hypoglycemic activity and, therefore, to be administered once a week. Novo Nordisk developed an ultra-long-acting basal insulin analogue (Icodec) as well as a fixed-dose combinations with GLP-1RA drugs (Icodec + semaglutide) [155]. On 21 March 2024, the Committee for Medicinal Products for Human Use (CHMP) of EMA adopted a positive opinion, recommending the granting of a marketing authorization for insulin Icodec, intended for the treatment of diabetes mellitus [166]. Icodec insulin has some modifications compared with human insulin: Tyr A14 and B16 are replaced by Glu and His, respectively, and Phe B25 by His, while Thr B30 is deleted; in addition, Lys B29 is acetylated with an icosanedioic acid. This variant shows a long half-life thanks to its stability since it is more protected from enzymatic degradation. Icodec binds reversibly to human serum albumin with an affinity ten times greater than determir and a minimal self-association; its slow absorption and ultra-long duration of action is a result of the formation of a circulating depot [154,155].

Eli Lilly and Company developed an ultra-long-acting insulin variant whose units are fused to the crystallizable (Fc) domain of a human immunoglobulin G2 fragment (insulin efsitora alfa). It presents several modifications with respect to the human variant; Ile A10 is replaced by a Thr; Tyr A14 and B16 by Asp and Glu, respectively; Phe B25 for His; and Gly units are included instead of Asn A21, Thr B27, Pro B28, Lys B29, and Thr B30 [167]. It has a long half-life and protection against degradation given by its greater stability and binding to immunoglobulin; its renal clearance is low, and it has a low self-association. Insulin efsitora is in an advanced stage of clinical development with several open phase III studies that compare its action, administered once a week, with the basal insulins degludec or glargine [154,156].

### 9.3. Prandial Insulins

The objective of prandial insulins is to reach the level of insulin secretion that occurs after eating food. They are short-acting insulins that allow the control of postprandial glycemic levels and constitute a complement to basal insulins (Table 12).

Patients with T2D may require insulin doses before meals without adding it to basal insulin. The recommended starting dose of insulin in mealtime is 4 units or 10% of the basal dose at each meal. With significant additions of prandial, particularly at dinner, have to be considered a reduction of the basal insulin dose.

Meta-analyses of trials comparing rapid-acting insulin analogues with regular human insulin in patients with T2D reported no significant differences in HbA1C or hypoglycemia.

Rapid human insulin (RHI) [152], also known as regular insulin, crystalline insulin, or neutral soluble human insulin, is obtained by recombinant DNA technology in Saccharomyces cerevisiae. Its action starts after 30 min; the maximum peak occurs between 2 and 4 h, and the maximum duration is 6–9 h. It can be prescribed every 6 h before the three meals together with delayed insulin at night, or before one or more of the three main meals, added to a basic regimen of delayed insulin in one or two doses.

Insulin aspart [168,169,170], is a recombinant insulin analogue with ultra-rapid action (5–20 min). It differs from human insulin by a single amino acid substitution at position B28, where Pro is substituted with Asp. This replacement reduces its tendency to form hexamers, thereby enhancing its absorption rate. Insulin aspart is produced using a genetically engineered strain of *Saccharomyces cerevisiae* (baker’s yeast). The maximum effect occurs approximately 1 to 3 h after dose and lasts 3 to 5 h. A long-acting insulin such as NPH insulin is usually also needed.

Glulisine [171,172,173] is a modified insulin of rapid action (about 15 min) obtained by recombinant DNA technology in Escherichia coli. It differs from native human insulin in Asn B3 substituted by Lys, and Lys B29 by Glu. These structural modifications decrease hexamer formation, stabilize monomers, and increase the rate of absorption and onset of action compared to human insulin. Unlike regular insulin, which must be administered about 15–30 min before meals, glulisine can be administered just before or even after eating, allowing greater schedule flexibility and adjusting the dose to the amount of carbohydrates ingested, which is especially useful in patients with T1D.

Lispro [174,175,176,177] is also a rapid insulin (15 min) produced by recombinant DNA technology using a non-pathogenic laboratory strain of Escherichia coli. It was the first commercially available insulin analogue. It differs from human insulin in Pro B28 replaced by Lys and Lys B29 by Pro. These modifications result in a reduced tendency to self-associate, which allows dissolution into a dimer and then into a monomer, which is absorbed more rapidly after subcutaneous injection compared to endogenous human insulin.

Diabetic patients who are treated with insulin inject the drug subcutaneously, a procedure that is not always comfortable and suitable for all patients. Suboptimal use of therapy is not uncommon, with omitted or inappropriate doses, which implies poor results and worsening adherence to treatment. This is why non-injectable insulin formulations are under continuous research. Two prandial insulins have been formulated and marketed: exubera insulin and technosphere, for inhaled administration.

Exubera is a dry powder mixture of recombinant human insulin, sodium citrate, sodium hydroxide, mannitol, and glycine, formulated in microspheres containing insulin monomers. It has rapid absorption and a duration of action of about 6 h, since microspheres reach the lung alveoli and dissolve at physiological pH [178] The efficacy observed in clinical studies and the favorable pulmonary safety profile allowed exubera to be the first inhaled insulin approved in 2006 for use in adult patients with diabetes mellitus in the USA and Europe. However, it was withdrawn from the market in 2007 (USA) and 2008 (Europe) due to low sales [179,180].

Insulin technosphere also consists of a dry powder mixture containing recombinant human insulin in self-assembly stable microspheres that absorb insulin monomers. Like exubera, it is quickly absorbed, and its action lasts between 1.5 and 4.5 h. It was authorized by the FDA in June 2014, and it is currently the only inhaled insulin preparation available in the USA for the treatment of postprandial hyperglycemia in adult patients living with diabetes mellitus [181,182].

### 9.4. Premixed Insulins

Premixed insulins were developed to improve patient convenience by reducing dosing frequency. They provide a basal and a prandrial component in a fixed combination, usually in concentrations of 100 IU/mL. These insulins are obtained by mixing an intermediate insulin and a regular insulin or with an ultrarapid analogue in the same injection device. These characteristics offer a mixed action that allows its use in two injections per day (Table 13).

The time to onset, peak, and duration are dictated by the short-acting component. Premixed insulins are identified by the percentage of each insulin/analogue component, with long-acting insulin first.

For the NPH human insulin/human insulin mixture, onset is around 30 to 45 min with a duration of action in 10–16 h. This insulin is usually dosed two to three times a day before breakfast and before dinner; this is convenient for patients who wish to limit the number of injections. However, responses to this insulin may be less reliable dose-to-dose than non-mixed insulins. Regular insulin can be precipitated in NPH insulin suspension, which may contribute to day-to-day variability in insulin action profiles [149].

Other mixed insulins available include rapid-acting analog insulin and protamine analog insulin. The protamine analogue delays absorption and facilitates a prolonged effect. Mixtures with a faster onset of action and a shorter maximum effect are administered immediately before meals, which may result in more reliable clinical effects and a reduction in postprandial hypoglycemia.

Premixed insulins are ideal when both basal and prandial insulin coverage are required but limiting the number of injections per day. In general, premixed insulins have a greater risk of hypoglycemia and require carbohydrate supplements in the periods between meals, which is generally associated with weight gain.

## 10. Conclusions

There is a great need to address the complications of a pathology as complex and multifactorial as diabetes, in the short and long term. Cardiovascular, renal, and metabolic effects are some of the alterations derived from this multiorgan and multifunctional state. The most promising therapy is the use of drug combinations (double or triple) to address the pathophysiological abnormalities derived from β-cell dysfunction and insulin resistance and thus achieve a first objective of normalizing plasma glucose levels.

The American Diabetes Association (ADA) and the European Association for the Study of Diabetes (EASD) recommend a patient-centered approach to choosing appropriate pharmacological treatment [3,5,10]. However, the first step before drug treatment is always lifestyle modifications that improve health.

The recommendations of both associations are as follows:

1. Metformin is the preferred initial pharmacological agent for the treatment of T2D. Once treatment is started, metformin should be continued as long as it is tolerated and is not contraindicated. Subsequently and based on progress, other agents, including insulin, should be added to metformin.
2. Long-term use of metformin may be associated with vitamin B12 deficiency, and periodic monitoring of vitamin B12 levels should be considered in patients treated with metformin, especially those with anemia or peripheral neuropathy.
3. Early introduction of insulin should be considered if there is evidence of ongoing catabolism (weight loss), if there are symptoms of hyperglycemia, or when HbA1c levels or blood glucose levels are very high.
4. Dual therapy should be considered in patients with newly diagnosed T2D who have HbA1c above their glycemic target.
5. A patient-centered approach is essential when selecting pharmacological agents. This decision-making process should consider a range of factors, including the presence of comorbidities, such as atherosclerotic cardiovascular disease, heart failure, or chronic kidney disease; the risk of hypoglycemia; the potential impact on body weight; treatment cost; the likelihood of adverse effects; and individual patient preferences.
6. Among T2D patients who have established cardiovascular disease, SGLT2 inhibitors or GLP-1 receptor agonists with demonstrated benefit in cardiovascular disease are recommended as part of the antihyperglycemic regimen.
7. Among patients with cardiovascular disease at high risk of heart failure or who have coexisting heart failure, SGLT2 inhibitors are recommended.
8. For patients with T2D and chronic kidney disease, the use of SGLT2 inhibitors or GLP-1 receptor agonists has been shown to reduce the risk of chronic kidney disease progression, cardiovascular events, or both; therefore, it should be considered.
9. In most patients who require the greatest hypoglycemic effect of an injectable medication, GLP-1 receptor agonists are preferred over insulin.
10. Treatment intensification should not be delayed for patients with T2M who do not meet treatment goals.
11. The medication regimen should be reevaluated at regular intervals (every 3 to 6 months) and adjusted as necessary to incorporate new patient factors.

In conclusion, although metformin remains the first-line agent for the treatment of T2D, the choice of a second-line agent depends on several factors, particularly the patient’s cardiovascular risk profile, weight, and renal function. Therefore, the ideal pharmacological treatment for diabetes control, although expected and desired, has in fact not been achieved so far, and physicians must consider not only the glycemic efficacy of the agent but also all other benefits balanced by potential adverse effects.

Experts believe that the three newer classes of antidiabetic agents, namely, SGLT2 inhibitors, GLP-1 receptor agonists, and DPP-4 inhibitors, should better demonstrate cardiovascular safety in addition to glycemic efficacy, but some of the SGLT2 inhibitors and GLP-1 receptor agonists have not only demonstrated non-inferiority but also superiority in clinical trials of cardiovascular outcomes, exhibiting promising prospects for the diabetes community.

Conventional insulin therapy for glycemic control warrants particular attention in this review, as it is often suboptimal and typically necessitates treatment intensification through either multiple daily injections or continuous subcutaneous infusion. However, adherence and persistence rates for insulin therapy remain lower than those for other antidiabetic medications. This situation could be attributed, among others, to two aspects: dosing more than once a day and injectable administration. Once-weekly dosing, by reducing the burden of injections, may help improve treatment adherence and persistence compared to multiple times-daily dosing. However, more research on once-weekly insulin dosing is needed to provide strong evidence of the impact of less frequent dosing on adherence and persistence of insulin therapy. On the other hand, considering that administration by subcutaneous injection contributes to suboptimal use, the suitability of non-injectable insulin administration continues to be intensively investigated.

The complexity of diabetes as a multifactorial disease strongly supports the rationale for simultaneously targeting multiple therapeutic pathways as a logical and promising strategy for its management and treatment. This approach involves the design and development of multitarget agents, characterized by complementary mechanisms of action acting on distinct receptors, potentially enhancing therapeutic efficacy through synergistic effects. However, such multimodality may also present drawbacks. Clinical development of certain multifunctional ligands has been discontinued due to adverse effects, which may arise from unbalanced or excessively potent pharmacological profiles. Therefore, the therapeutic potential of compounds capable of modulating multiple signaling pathways demands further rigorous evaluation. Advancements in the understanding of the molecular basis and signaling mechanisms underlying diabetes are expected to facilitate the development of novel therapeutic strategies, including personalized medicine approaches.

## Figures and Tables

**Table 1:** Types of diabetes [2,6,7,8].

Types of Diabetes	Description
Type 1	Typically results from autoimmune destruction of pancreatic β cells, leading to a complete lack of insulin production. Frequently diagnosed in childhood or adolescence, though it can appear at any age.
Type 2	Most prevalent form. Characterized by a gradual decline in β-cell function and insulin production, typically occurring in the context of insulin resistance and metabolic syndrome. Frequently linked to excess body weight and obesity.
**Hybrid Forms of Diabetes**	
Autoimmune diabetes of slow evolution in adults	Similar to type 1. It progresses slowly in adults. It has features of metabolic syndrome, has a single GAD (Glutamate Decarboxylase) autoantibody, and retains increased β-cell functionality.
Diabetes with a tendency to ketosis	Characterized by episodes of ketosis and initial insulin deficiency, though long-term insulin therapy may not be required. Ketosis tends to occur frequently, but the condition is not driven by autoimmune mechanisms.
**Other Specific Types of Diabetes**	
Monogenic diabetes	Caused by specific genetic mutations. Monogenic defects in the functionality of β cells. This form of diabetes presents diverse clinical profiles, each requiring tailored therapeutic approaches. Some cases emerge during the neonatal stage, while others appear in early adulthood. It is associated with monogenic mutations affecting insulin signaling, leading to pronounced insulin resistance in the absence of obesity. The condition arises when pancreatic β cells are unable to adequately compensate for the body’s insulin resistance.
Exocrine pancreas diseases	Cystic fibrosis and pancreatitis.
Endocrine disorders	Diseases with excess of insulin antagonist hormone secretion.
Induced by drugs/chemicals	Certain medications and chemical agents can interfere with insulin production or lead to the destruction of pancreatic β cells.
Infection-related diabetes	Viruses associated with the direct destruction of β cells.
Rare specific forms of autoimmune diabetes	Linked to uncommon autoimmune disorders that may contribute to the development of diabetes.
Other genetic syndromes related with diabetes	Numerous genetic syndromes and chromosomal anomalies are associated with an elevated risk of developing diabetes.
Unclassified diabetes	Cases of diabetes that do not clearly align with established classifications. It is intended as a provisional designation, particularly useful in the early stages after diagnosis, when a definitive categorization is not yet possible.
**Hyperglycemia first detected during pregnancy**	
In pregnancy	Type 1 or type 2 diabetes first diagnosed during pregnancy.
Gestational	Hyperglycemia below diagnostic levels for diabetes in pregnancy.

**Table 2:** Criteria based on glycemia for the diagnosis of diabetes.

Parameter	Normal *	Prediabetes	T2D
Hemoglobin A1c (HbA1c)	<5.7% ^‡^ <6.0% ^§^	5.7–6.4% ^‡^ 6.0–6.4% ^§^	≥6.5%
Fasting plasma glucose (FPG)	100 mg/dL ^‡^ <110 mg/dL ^§^	100–125 mg/dL ^‡^ 110–125 mg/dL ^§^	≥126 mg/dL
2 h plasma glucose value during 75g oral glucose tolerance test (OGTT)	<140 mg/dL	140–199 mg/dL	≥200 mg/dL

* Normal glucose metabolism. ^‡^ American Diabetes Association [10]. ^§^ World Health Organization [7].

**Table 3:** Sulfonylureas. Dosage and pharmacokinetic characteristics [40].

Drug	Starting Dose (Maximum Daily Dose)	T_max_ (h)	Duration of Action (h)	Metabolism	Elimination
**First Generation**					
Tolbutamide	250 mg 1–3/day (3000 mg)	3–4	6–12	Inactive metabolites	Urine
Chloropropamide	100 mg/day (750 mg)	2–4	≥24	Active metabolites	Urine
Tolazamida	100 mg/day (1000 mg)	3–4	12–24	Active & inactive metabolites	Urine 85%, Feces 7%
**Second Generation**					
Glibenclamide (Glyburide)	2.5 mg/day (20 mg in 1–2 doses)	2–4	16–24	Inactive or weakly active metabolites	Urine 50% Feces 50%
Glipizide	2.5 mg/day (20 mg in 1–2 doses)	1–3 (IR) 6– 12 (ER)	12–24	Inactive metabolites	Urine 90% Feces 10%
Gliquidone	15 mg/day (180 mg in 2–3 doses)	2–3	8–10	Inactive metabolites	Urina 5% Feces 95%
Gliclazide	40 mg/day (320 mg in 2 doses) 30–120 mg/day MR	4–6 6–12 (MR)	10–24 ≥24 (MR)	Inactive metabolites	Urine 60–70% Feces 10–20%
Glimepiride	1 mg/day (6 mg)	2–3	≥24	Inactive and active metabolites	Urine 60% Feces 40%

ER: Extended release; IR: Immediate release; MR: Modified release; T_max_: Time to maximum plasma concentration.

**Table 4:** Effects observed of GLP-1 and GLP-1 RA on various organs and tissues [57].

Organ/Tissue	At Physiological Levels of GLP-1 in Clinical Studies	After Treatment with GLP-1 RA	In Preclinical Studies
Adipose			*Increase* Lipolysis Glucose uptake
Brain	*Increase* Satiety *Decrease* Appetite		
Gastrointestinal	*Decrease* Gastric emptying Acid secretion		
Heart	*Increase* Endothelial function	*Increase* Heart rate Myocardial contractility Diastolic function Cardiprotection *Decrease* Blood pressure	
Kidney	*Increase* Natriuresis		
Muscle	*Increase* Glycogen synthesis Glucose oxidation		
Pancreas	*Increase* Insulin secretion *Decrease* Glucagon secretion		*Increase* β cell proliferation

GLP-1 RA: GLP-1 receptor agonists.

**Table 6:** Summary of the most significant clinical outcomes with GLP-1-RA [66].

Drug	Relative Efficacy		MACE Composite Outcome [b]
	Reduction of HbA1c [a]	Reduction of Body Weight [a]	
Dulaglutide 1/week [d] SC	++	++	Superior to placebo
Exenatide 2/day SC	+	+(+)	No CVOT
Exenatide XR 1/week SC	+	+	Not inferior to placebo
Lixisenatide 1/day SC	+	+	Not inferior to placebo
Liraglutide 1/day [c] SC	++	++	Superior to placebo
Semaglutide 1/week [c] SC	+++	+++	Superior to placebo
Oral semaglutide 1/day	++(+)	++(+)	Not inferior to placebo

[a] Estimates based on clinical judgment and data from randomized controlled clinical studies of GLP-1 RA as monotherapy or as a single addition to antihyperglycemic therapies. [b] Based on results of CVOT with a primary outcome of MACE (first events of cardiovascular death, nonfatal myocardial infarction, or nonfatal stroke). [c] FDA approved to reduce MACE in adults with T2D who have established CVD. [d] FDA-approved to reduce MACE risk in adults with T2D who have established CVD or multiple cardiovascular risk factors. CVD: cardiovascular disease; CVOT: cardiovascular outcomes trial; HbA1c: glycosylated hemoglobin; MACE: major cardiovascular adverse effects; SC Subcutaneous; XR: extended release.

**Table 7:** Pharmacokinetic profile of frequently used DPP-4 inhibitors. All given orally [58].

DPP-4 Inhibitor	Dose	t_1/2_	BA	PPB	Metabolism	Elimination
Alogliptin	25 mg 1/day	20 h	100%	20–30%	Minimal	Renal (>70%)
Linagliptin	5 mg 1/day	~12 h (effective) >100 h (terminal)	~30%	>90%	Minimal	Hepatic (Renal < 7%)
Sitagliptin	5 mg 1/day	2.5 h (drug) 3 h (metabolite)	~75%	N	Hydrolysis (CYP 3A4 and 3A5) to form active metabolite	Metabolism (drug) Renal (metabolite)
Sitagliptin	100 mg 1/day	12.5 h	~87%	18%	Minimal	Renal (>80%)
Vildagliptin	50 mg 2/day	2 h	85%	~9%	Hydrolysis (CYP independent) to form inactive metabolite	Metabolism (drug) Renal (metabolite)

BA: Absolute bioavailability; CYP: Cytochrome P450; N: Negligible; PPB: Binding to plasma proteins.

**Table 8:** Cardiovascular (CV) safety as MACE (hazard ratio, primary outome) and hospitalization risk for heart failure (hazard ratio) in clinical studies of some dipeptidyl peptidase-4 inhibitors (DDP4i) [58].

Compound	CV Safety	Hospitalization Risk
Aligliptin	0.96 vs. Placebo	1.07 (*p =* 0.66) vs. Placebo
Linagliptin	1.02 vs. Placebo 0.98 vs. Glimepiride	0.90 (*p =* 0.26) vs. Placebo 1.21 (*p =* 0.18) vs. Glimepiride
Saxagliptin	1 vs. Placebo	1.27 (*p <* 0.007) vs. Placebo
Sitagliptin	0.98 vs. Placebo	1.00 (*p* = 0.98) vs. Placebo
Vildagliptin	No data	No data

MACE. major adverse cardiovascular events.

**Table 9:** Pharmacokinetic parameters of therapeutically used SGLT2 inhibitors. All drugs are metabolized in the liver through glucuronidation and excreted via the renal route [94].

Parameter	Canagliflozin	Dapagliflozin	Empagliflozin	Ertugliflozin
Oral dose (mg)	100–300	5–10	10–25	5–15
Half-life (hours)	10.6–13.1	12.9	12.4	16.6
Bioavailability (%)	65	78	60	100
Volume of distribution (L)	83.5	118	73.8	~2000 times
Plasma protein binding (%)	98	91	86.2	93.6
Activity as SGLT2i vs. SGLT1i	~250 times	~1200 times	~2500 times	~2000 times

**Table 10:** Use of SGLT2 inhibitors in patients with T2D or heart failure (HR) based on renal function, considered as estimated glomerular filtration rate eGFR (mL/min/1.73 m^2^) [92].

Drug	SD (mg)	MD (mg)	*T2D*		*HF*	
			*eGFR* *Limit*	*Dose (mg)*	*eGFR* *Limit*	*Dose (mg)*
Canagliflozin	100	100–300	60 30–59 <30	Max 300 Max 100 Max 100 in patients already treated with the drug. If not, do not initiate		
Dapagliflozin	10	10	≥45 <45 <25	10 Additional hypoglycemic treatment 10 mg in patients already treated with the drug; if not, contraindicated	≥25 <25	10 mg 10 mg in patients already treated with the drug; if not, do not initiate
Empagliflozin	10	10	≥60 45–59 <45	Max 25 Max 20 mg, only if before treatment eGFR ≥60 Contraindicated	≥20 <20	10 mg Contraindicate
Ertugliflozin	5	5–15	<60 <45	Do not initiate Contraindicated		

SD: starting dose, MD: maintenance dose.

**Table 11:** Basal insulins [152,153,154]. First and second generation administered as subcutaneous injections in presentations available as vials, cartridges, or prefilled-pen. Third-generation developments focused on oral, inhaled, and ultra-insulins.

Insulin	Type	Concentration Dose	Action Onset	Time to Peak Action	Duration Action
***First generation***					
NPH	Intermediate	40 and 100 UI/mL	1–4 h	4–12 h	12–24 h
Detemir	Long	100 UI/mL	1–2 h	4–8 h	12–24 h
Glargine U100	Long	100 UI/mL	1–4 h	8–12 h	5–24 h
***Second generation***					
Glargine U300	Ultra-long	300 UI/mL	2–6 h	12 h & no peak	30–36 h
Degludec	Ultra-long	100 and 200 UI/mL	0.5–2 h	9 h & no peak	>42 h
***Third generation. Once weekly insulins. Under development***					
Icodec [155]	Ultra-long	700 UI/mL	0.5–2 h	no peak	196 h
Efsitora alfa [156]	Ultra-long	35 units/mg		3–5 days	408 h

NPH: neutral protamine Hagedorn insulin, also known as isophane insulin [157].

**Table 12:** Prandial insulins [154]. First and second generation administered as subcutaneous injections in presentations available as vials, cartridges, or prefilled-pen. Third-generation developments formulated for inhaled administration.

Insulin	Type	Concentration Dose	Action Onset	Time to Peak Action	Duration Action
***First generation***					
RHI	Intermediate	100 UI/mL	0.5–1 h	2.4–4 h	5–8 h
Aspart	Rapid	100 UI/mL	5–20 min	1–3 h	3–5 h
Glulisine	Rapid	100 UI/mL	9–20 min	1–3 h	3–5 h
Lispro 100 UI	Rapid	100 UI/mL	9–20 min	1–3 h	3–5 h
***Second generation***					
Lispro 200 UI	Rapid	200 UI/mL	9–20 min	1–3 h	3–5 h
***Third generation. Inhaled insulins***					
Exubera	Rapid	1 and 3 mg	10–20 min	2 h	6 h
Tecnosphere	Rapid	4, 8, and 12 UI	12 min	35–55 min	1.5–4.5 h

RHI: regular human insulin.

**Table 13:** Premixed insulins [154]. All of them are of biphasic action (basal + prandial). Administered as subcutaneous injections in presentacionas available as vials, cartridges, or prefilled-pen.

Mixture	Concentration Dose	Action Onset	Time to Peak Action	Duration Action
***First generation***				
NPH + RHI	100 UI	0.5–1 h	2–5 h	10–16 h
Protaminated aspart + Aspart	1000 UI	5–30 min	1–12 h	15–18 h
Protaminated lispro + Lispro	1000 UI	10–15 min	1–12 h	10–16 h
***Second generation***				
RHI	500 UI	<15 min	4–8 h	13–24 h
***Third generation***				
Degludec + Aspart	100 UI	10–20 min	0.5–1.5 h	>24 h

NPH: neutral protamine Hagedorn insulin, also known as isophane insulin [157]. RHI: regular human insulin.

## Data Availability

Not applicable.

## Abbreviations

The following abbreviations are used in this manuscript:

2-h PG	2 h plasma glucose
A1C or A1c or HbA1c	Glycosylated hemoglobin
AGI	α-Glucosidase inhibitors
AMPK	AMP-activated protein kinase
AMP	Adenosine monophosphate
AR	Aldose reductase
ASCVD	Atherosclerotic cardiovascular disease
AUC	Area under the curve
BMI	Body mass index
CAD	Diabetic ketoacidosis
CHMP	Committee for Medicinal Products for Human Use of EMA
CKD	Chronic kidney disease
CV	Cardiovascular
CVD	Cardiovascular disease
CVOT	Cardiovascular outcome trial
Cyt	Cytochrome
DAPA-HF	Dapagliflozin and Prevention of Adverse Events in Heart Failure
DDP-4	Dipeptidyl peptidase-4
DDP-4i	Dipeptidyl peptidase-4 inhibitor
DKA	Diabetic ketoacidosis
DKD	Diabetic kidney disease
DM	Diabetes mellitus
DNL	De novo lipogenesis
ECV	Cardiovascular disease
EMA	European Medicines Agency
ER	Extended release
eGFR	Estimated glomerular filtration rate
FDA	US Food and Drug Administration
FPG	Fasting plasma glucose
GADA	Glutamate decarboxylase autoantibodies
GI	Gastrointestinal
GIP	Glucose-dependent insulinotropic polypeptide
GLP-1	Glucagon like peptide-1
GLP-1 RA	Glucagon-like peptide-1 receptor agonist
GLUT	Glucose transporter protein
GSV	GLUT4 storage vesicles
HbA1c	Glycosylated hemoglobin
HDL	High-density lipoproteins
HF	Heart failure
HFrEF	Heart Failure with reduced ejection fraction
hHF	Hospitalization for heart failure
HIV	Human insufficiency virus
HOMA2-B	Homeostatic Model for Assessing β-Cell Functionality
HOMA2-IR	Homeostatic Model for Assessing Insulin Resistance
IDF	International Diabetes Federation
IFG	Impaired fasting glucose
IGT	Impaired glucose tolerance
IL-6	Interleukin 6
INSR	Insulin receptor
IR	Insulin resistance
IR	Immediate release
LADA	Latent autoimmune diabetes in adults
LDL	Low-density lipoproteins
LVEF	Left ventricular ejection fraction
LVH	Left ventricular hypertrophy
MACE	Major cardiovascular adverse effects
MARD	Moderate age-related diabetes
MR	Modified release
mRNA	Messenger RNA
MOD	Mild obesity-related diabetes
NCE	New chemical entity
NEFA	Fatty acids in esterified
NPH	Neutral protamine Hagedorn
OGTT	Oral glucose tolerance test
OXM	Oxyntomodulin
PIP	Phosphatidylinositol phosphate
PPAR	Peroxisome proliferator-activated receptors (PPAR)
PPB	Binding to plasma proteins
PTP1B	Protein tyrosine phosphatase 1B
RHI	Regular human insulin
RR	Risk ratio
SAID	Severe autoimmune diabetes
SGLT1	Sodium-glucose linked transporter type 1
SGLT2	Sodium-glucose linked transporter type 2
SGLT2i	Sodium-glucose linked transporter type 2 inhibitor
SIDD	Severe insulin-deficient diabetes
SIRD	Severe insulin-resistant diabetes
SmPC	Summary of product characteristics
SU	Sulfonylurea
t_1/2_	Half-life of elimination
T1D	Type 1 diabetes
T2D	Type 2 diabetes or type 2 diabetes mellitus
T_max_	Time to maximum plasma drug concentration
TNF	Tumor necrosis factor
TZD	Thiazolidinedione
UACR	Urine albumin-creatinine ratio
VLDL	Very low-density lipoproteins
wk	Week
XR	Extended release
